# Measurable Residual Disease Assessed by Flow-Cytometry Is a Stable Prognostic Factor for Pediatric T-Cell Acute Lymphoblastic Leukemia in Consecutive SEHOP Protocols Whereas the Impact of Oncogenetics Depends on Treatment

**DOI:** 10.3389/fped.2020.614521

**Published:** 2021-02-05

**Authors:** Nerea Vega-García, Sara Perez-Jaume, Elena Esperanza-Cebollada, Clara Vicente-Garcés, Montserrat Torrebadell, Antonio Jiménez-Velasco, Margarita Ortega, Marta Llop, Lorea Abad, José Manuel Vagace, Alfredo Minguela, Marta Pratcorona, Joaquín Sánchez-Garcia, Clara B. García-Calderón, María Teresa Gómez-Casares, Estela Martín-Clavero, Adela Escudero, Marta Riñón Martinez-Gallo, Luz Muñoz, María Rosario Velasco, Marina García-Morin, Albert Català, Antonia Pascual, Pablo Velasco, José Mª. Fernández, Alvaro Lassaletta, José Luis Fuster, Isabel Badell, Águeda Molinos-Quintana, Antonio Molinés, Pilar Guerra-García, Antonio Pérez-Martínez, Miriam García-Abós, Reyes Robles Ortiz, Sandra Pisa, Rosa Adán, Cristina Díaz de Heredia, José Luis Dapena, Susana Rives, Manuel Ramírez-Orellana, Mireia Camós

**Affiliations:** ^1^Haematology Laboratory, Hospital Sant Joan de Déu, University of Barcelona, Barcelona, Spain; ^2^Developmental Tumor Biology Group, Leukemia and Other Pediatric Hemopathies, Institut de Recerca Sant Joan de Déu, Barcelona, Spain; ^3^Developmental Tumour Biology Laboratory, Institut de Recerca Hospital Sant Joan de Déu Barcelona, Barcelona, Spain; ^4^Centro de Investigación Biomédica en Red de Enfermedades Raras (CIBERER), Instituto de Salud Carlos III, Madrid, Spain; ^5^Haematology and Hemotherapy Laboratory, Hospital Carlos Haya, Málaga, Spain; ^6^Cytogenetics Unit, Hematology Department, Hospital Vall d'Hebron, Barcelona, Spain; ^7^Molecular Biology Unit, Clinical Analysis Service, La Fe University and Polytechnic Hospital, Valencia, Spain; ^8^Centro de Investigación Biomédica en Red - Cáncer (CIBERONC CB16/12/00284), Madrid, Spain; ^9^Paediatric Hemato-Oncology Laboratory, Hospital Niño Jesús, Madrid, Spain; ^10^Haematology Laboratory, Hospital Materno Infantil, Badajoz, Spain; ^11^Immunology Service, Clinic University Hospital Virgen de la Arrixaca (HCUVA) and Instituto Murciano de Investigación Biosanitaria (IMIB), Murcia, Spain; ^12^Haematology Laboratory, Hospital de la Santa Creu i Sant Pau, Barcelona, Spain; ^13^Hematology Department, Hospital Reina Sofía, IMIBIC, UCO, Córdoba, Spain; ^14^Instituto de Biomedicina de Sevilla (IBIS/Consejo Superior de Investigaciones Científicas (CSIC)/Centro de Investigación Biomédica en Red - Cáncer (CIBERONC)), Hospital Universitario Virgen del Rocío, Universidad de Sevilla, Seville, Spain; ^15^Biology and Molecular Haematology and Hemotherapy Service, Hospital Universitario de Gran Canaria Doctor Negrín, Las Palmas de Gran Canarias, Spain; ^16^Haematology-Cytology Department, Hospital Universitario 12 de Octubre, Madrid, Spain; ^17^Translational Research in Pediatric Oncology Hematopoietic Transplantation and Cell Therapy, Institute of Medical and Molecular Genetics (INGEMM), Hospital La Paz Institute for Health Research (IdiPAZ), Madrid, Spain; ^18^Immunology Laboratory, Hospital de Cruces, Bilbao, Spain; ^19^Haematology Laboratory, Hospital Parc Taulí, Sabadell, Spain; ^20^Haematology Department, Hospital Virgen de la Salud, Toledo, Spain; ^21^Paediatric Hematology Unit, Hospital General Universitario Gregorio Marañón, Madrid, Spain; ^22^Paediatric Hematology and Oncology Departments, Hospital Sant Joan de Déu, University of Barcelona, Barcelona, Spain; ^23^Haematology Department, Hospital Carlos Haya, Málaga, Spain; ^24^Pediatric Hematology and Oncology Department, Hospital Universitari Vall d'Hebron, Barcelona, Spain; ^25^Haematology and Oncology Department, Hospital de La Fe, Valencia, Spain; ^26^Haematology and Oncology Department, Hospital Niño Jesús, Madrid, Spain; ^27^Paediatric Oncohematology Department, Clinic University Hospital Virgen de la Arrixaca (HCUVA) and Instituto Murciano de Investigación Biosanitaria (IMIB), Murcia, Spain; ^28^Paediatric Hematology Department, Hospital de la Santa Creu i Sant Pau, Barcelona, Spain; ^29^Unit of Hematology and Hemotherapy, H.U. Materno Infantil de Canarias, Canarias, Spain; ^30^Paediatric Hemato-Oncology Department, Hospital Universitario 12 de Octubre, Madrid, Spain; ^31^Department of Pediatric Hemato-Oncology and Stem Cell Transplantation, La Paz University Hospital, Madrid, Spain; ^32^Pediatric Onco-Hematology Department, Hospital Universitario Donostia, Donostia, Spain; ^33^Pediatric Onco-Hematology Department, Complejo Hospitalario de Navarra, Navarra, Spain; ^34^Paediatric Hematology Department, Hospital Parc Taulí, Sabadell, Spain; ^35^Haematology and Oncology Department, Hospital de Cruces, Bilbao, Spain

**Keywords:** measurable (minimal) residual disease, T-cell acute lymphoblastic leukemia, oncogenetics, NOTCH1, flow cytometry, pediatrics, risk-factors

## Abstract

Robust and applicable risk-stratifying genetic factors at diagnosis in pediatric T-cell acute lymphoblastic leukemia (T-ALL) are still lacking, and most protocols rely on measurable residual disease (MRD) assessment. In our study, we aimed to analyze the impact of *NOTCH1, FBXW7, PTEN*, and *RAS* mutations, the measurable residual disease (MRD) levels assessed by flow cytometry (FCM-MRD) and other reported risk factors in a Spanish cohort of pediatric T-ALL patients. We included 199 patients treated with SEHOP and PETHEMA consecutive protocols from 1998 to 2019. We observed a better outcome of patients included in the newest SEHOP-PETHEMA-2013 protocol compared to the previous SHOP-2005 cohort. FCM-MRD significantly predicted outcome in both protocols, but the impact at early and late time points differed between protocols. The impact of FCM-MRD at late time points was more evident in SEHOP-PETHEMA 2013, whereas in SHOP-2005 FCM-MRD was predictive of outcome at early time points. Genetics impact was different in SHOP-2005 and SEHOP-PETHEMA-2013 cohorts: *NOTCH1* mutations impacted on overall survival only in the SEHOP-PETHEMA-2013 cohort, whereas homozygous deletions of *CDKN2A*/*B* had a significantly higher CIR in SHOP-2005 patients. We applied the clinical classification combining oncogenetics, WBC count and MRD levels at the end of induction as previously reported by the FRALLE group. Using this score, we identified different subgroups of patients with statistically different outcome in both Spanish cohorts. In SHOP-2005, the FRALLE classifier identified a subgroup of high-risk patients with poorer survival. In the newest protocol SEHOP-PETHEMA-2013, a very low-risk group of patients with excellent outcome and no relapses was detected, with borderline significance. Overall, FCM-MRD, WBC count and oncogenetics may refine the risk-stratification, helping to design tailored approaches for pediatric T-ALL patients.

## Introduction

Compared to the particularly good outcome of pediatric patients with B-cell precursor acute lymphoblastic leukemia (BCP-ALL), T-ALL patients still do worse, and 15–20% of pediatric and 40% of adult T-ALL patients relapse. Importantly, relapsed T-ALL is often highly resistant, and such patients present a dismal prognosis (1, 2). Hence, prevention of relapse is imperative, and patients are allocated to intensive and often very toxic therapeutic regimes with short- and long-term side effects. A precise risk stratification of T-ALL patients would allow to dynamically adjust the intensity of treatment, helping to balance the risks of relapse and treatment-related toxicity. Current therapeutic protocols aim to tailor treatment by applying less intensive therapy to low-risk patients, thus reducing the risk of treatment-related toxicity, while reserving intensive therapy to high-risk patients. In contrast to BCP-ALL, the new biological insights in T-ALL have scarcely been incorporated to current protocols, and risk stratification relies mainly on response to treatment. Thus, although white blood cell (WBC) count and early response to prednisone at day 8 are used, measurable residual disease (MRD) quantification post-remission remains the most important risk factor (3–5). T-ALL patients show a slower blast clearance compared to BCP-ALL, and the MRD detection at a late time point (day 78) may define better the risk of relapse in T-ALL patients than earlier evaluation at end of induction (day 33) in some therapeutic protocols (6). The MRD gold standard method is real-time quantitative polymerase chain reaction for immunoglobulin and T-cell receptor clonality (IG-TR PCR-MRD) (6–8). Flow-cytometry MRD measurement (FCM-MRD), based on the leukemia-associated immunophenotype, is also used for risk stratification in some collaborative groups and provides reliable identification of patients eligible for reduced-intensity therapy in T-ALL and BCP-ALL (9–12). However, even in the setting of MRD-oriented protocols, some T-ALL patients relapse, and it is important to identify additional prognostic factors.

Multiple genetic abnormalities have been identified in T-ALL cases (13, 14), including proto-oncogene activation, tumor-suppressor gene deletions and constitutive activation of the NOTCH1 pathway, present in 40–60% of patients (15–17). The prognostic impact of *NOTCH1* and *FBXW7* mutations is controversial; it has been usually associated with favorable prognosis (18–20), but other studies have found no significant effect (21, 22), and some authors have reported that mutant *FBXW7* independently predicts an inferior survival (23). Recent studies indicate that the impact of *NOTCH1/FBXW7* mutations could be modulated by concomitant mutations in *PTEN* and *RAS* genes (3). *PTEN* loss through mutation or genomic deletion occurs in up to 35% of T-ALL pediatric patients, and ~10% harbor *N-* or *K-RAS* mutations. Although these abnormalities are usually associated with unfavorable prognosis, their impact in pediatric T-ALL patients is still unclear (24–27). The Group for Research in Adult Acute Lymphoblastic Leukemia (GRAALL) validated in adult T-ALL patients the clinical utility of a new oncogenetic classifier based on the mutational status of *NOTCH1/FBXW7/PTEN/RAS* (*NFPR*) genes (28). In this model, the presence of *NOTCH1* and *FBXW7* mutations without lesions involving *PTEN/RAS* defined the genetic low-risk subgroup (gLoR), and other genotypes were allocated to the genetic high-risk group (gHiR). In contrast, in a study in 145 pediatric T-ALL patients treated with the MRC UKALL2003 trial, neither *PTEN* nor *RAS* significantly impacted on outcome, and none of these mutations changed the highly favorable outcome of patients with double *NOTCH1/FBXW7* mutations (29). Recently, the FRALLE group (French Acute Lymphoblastic Leukemia Study Group) suggested a new classifier based on WBC count, MRD by IG-TR PCR-MRD and the oncogenetic mutational status, which improved prediction of the relapse risk for their pediatric T-ALL patients (3).

Copy number alterations (CNA) have been reported as prognostic markers, both in BCP-ALL and in T-ALL (30, 31). *CDKN2A/B* loci deletion is present in up to 70% of T-ALL patients. In adult T-ALL, *CDKN2A/B* homozygous deletion (*CDKN2A/B*^*homo*^*)* was associated with a favorable prognosis only within the mature/cortical group (32), while other studies showed a correlation with poor survival or no association with outcome (31, 33). Overall, the role of oncogenetics as risk factors in T-ALL is controversial and seems to differ according to the treatment protocol.

We present a retrospective collaborative study of the Biological Committee of the Leukemia Group of the SEHOP (Spanish Society of Pediatric Hematology and Oncology) on a series of 199 pediatric T-ALL patients treated according to SEHOP and PETHEMA (Programa para el Estudio de la Terapéutica en Hemopatías Malignas) Cooperative Groups. In both protocols, we aimed to: (1) confirm the clinical impact of FCM-MRD measurement, (2) assess the prognostic significance of MRD at late time points, and (3) test whether the previously reported oncogenetic classifier by the FRALLE group could refine the risk stratification in our patients receiving BFM-based protocols.

## Materials and Methods

### Study Design, Cohort of Patients, and Therapeutic Protocols

The study design is shown in Supplementary Figure 1. This is a retrospective study, designed to include pediatric patients diagnosed with T-ALL in Spain with available biological material. The participation in the study was offered to all the Spanish centers belonging to the SEHOP Group of Leukemias. DNA samples from 26 different Spanish centers were sent to Hospital Sant Joan de Déu to perform the oncogenetic studies. The referring centers provided data about clinical and basic biological characterization of their patients at diagnosis and the levels of MRD during follow-up. The clinic-biological data of the patients included in our study are detailed in Table 1 and Supplementary Table 1. A total of 199 pediatric T-ALL patients treated according to SEHOP and PETHEMA Cooperative Groups from 1998 to 2019 were collected. We analyzed the molecular features of 189 patients, after excluding cases with T-cell lymphoblastic lymphoma, and patients with insufficient sample or no clinical data. For the survival analysis, we excluded those patients treated as per PETHEMA protocols, as they had been treated heterogeneously according to different therapeutic regimes. The 142 patients selected for the survival analyses were treated according to the consecutive protocols SHOP-2005 (*n* = 51) and SEHOP-PETHEMA-2013 (*n* = 91). These patients were representative of the whole SHOP-2005 and SEHOP-PETHEMA-2013 population in terms of clinical characteristics at diagnosis and outcome and only differed in a longer follow-up for the patients included in the study (Supplementary Table 2).

**Table 1:** Clinical and molecular features of patients included in SEHOP-PETHEMA-2013 and SHOP-2005 protocols for the survival analyses.

	**SHOP-2005 (*n* = 51)**	**SEHOP-PETHEMA-2013 (*n* = 91)**	***p*-value**
Sex	*n* = 51	*n* = 91	
Male	38 (74.5%)	67 (73.6%)	1.00
Female	13 (25.5%)	24 (26.4%)	
Age, years, median [range min; max]	8.72 [1.69; 19.0]	7.49 [1.43; 16.0]	0.30
Age	*n* = 51	*n* = 91	0.37
<10 years	29 (56.9%)	60 (65.9%)	
≥10 years	22 (43.1%)	31 (34.1%)	
WBC count, median [range min; max]	56.9 [1.00; 675]	72.2 [1.90; 897]	0.51
WBC count	*n* = 51	*n* = 89	0.88
<200 × 10^9^/L	37 (72.5%)	67 (75.3%)	
≥200 × 10^9^/L	14 (27.5%)	22 (24.7%)	
CNS	*n* = 51	*n* = 83	
1	47 (92.2%)	55 (66.3%)	
2	4 (7.84%)	12 (14.5%)	0.001
3	0 (0.00%)	16 (19.3%)	
Phenotype	*n* = 37	*n* = 83	
Cortical	21 (56.8%)	41 (49.4%)	0.58
Other	16 (43.2%)	42 (50.6%)	
*NOTCH1*	*n* = 47	*n* = 90	
Wild type	32 (68.1%)	57 (63.3%)	0.72
Mutated	15 (31.9%)	33 (36.7%)	
*FBXW7*	*n = 51*	*n = 89*	
Wild type	42 (82.4%)	77 (86.5%)	0.68
Mutated	9 (17.6%)	12 (13.5%)	
*PTEN*	*n* = 51	*n* = 89	
Wild type	46 (90.2%)	79 (88.8%)	1.00
Abnormality	5 (9.80%)	10 (11.2%)	
*N/K-RAS*	*n* = 51	*n* = 89	
Wild type	45 (88.2%)	84 (94.4%)	0.21
Mutated	6 (11.8%)	5 (5.62%)	
**Oncogenetics**	*n* = 44	*n* = 82	
gLoR	15 (34.1%)	31 (37.8%)	0.83
gHiR	29 (65.9%)	51 (62.2%)	
**Fusion-gene**	*n* = 41	*n* = 81	
*NUP214-ABL1*	3 (7.32%)	2 (2.47%)	0.23
*STIL-TAL1*	6 (14.6%)	12 (14.8%)	0.36

*WBC, white blood cell; CNS, central nervous system; meaning 1: no blasts in CNS, 2: blasts in CNS with ≤ 5 WBC/μl and/or traumatic lumbar puncture, and 3: blast in CNS with <5 WBC/μl or CNS symptoms*.

SHOP-2005 (2005–2013) and SEHOP-PETHEMA-2013 (2013-present) are two consecutive therapeutic protocols BFM (Berlin-Frankfurt-Münster)-inspired. The former has been previously described (5), and the full details of SEHOP-PETHEMA-2013 protocol are provided in the Supplementary Material. Patients with T-ALL were not eligible for standard risk group stratification in either protocol. To summarize the main differences between both protocols, in the newer SEHOP-PETHEMA-2013: (1) prednisone response was incorporated as initial stratification criteria; (2) an Induction IB course was added; (3) craniospinal irradiation was omitted and increased number of doses of triple intrathecal therapy were used instead; (4) there was a more intensive use of asparaginase, with more doses of asparaginase, use of PEG-asparaginase and longer periods of asparagine depletion; (5) there were more restrictive indications of allogeneic stem cell transplantation (allo-SCT), mainly based on FCM-MRD criteria, and (6) eight-colors FCM-MRD was performed in reference laboratories, in contrast with decentralized 4-color FCM-MRD in SHOP-2005 (see below).

### FCM Characterization and MRD Assessment

The immunophenotyping by FCM was performed in each laboratory using the combination of antibodies and panels detailed for each protocol in the Supplementary Material. Briefly, in SHOP-2005 protocol, 4-color panels were used in each center for identification and monitoring of leukemia-associated immunophenotypes (LAIPs), according to the guidelines of group EGIL (34). For residual disease analysis, either patient-tailored antibody combinations or those employed at diagnosis were used. In this protocol, MRD levels >0.1% were considered positive and used for clinical decisions. However, lower values were reviewed and recorded for the analysis in the present study. In SEHOP-PETHEMA-2013 protocol, an 8-colors panel according to EuroFlow consortium was recommended at diagnosis (35), and FCM-MRD was centralized in 10 reference laboratories. For MRD detection down to the 0.01% level (with a required resolution of at least 20 events to refer a sample as positive), an optimum of 500,000 nucleated cells needed to be acquired. MRD was assessed at day 15, end of induction (time point 1, TP1) and after the second course of chemotherapy, around day 78 (timepoint 2, TP2). In this protocol, MRD levels >0.01% were considered positive, and the thresholds considered for clinical decisions varied according to the timepoint. Thus, patients were stratified to high-risk arm if FCM-MRD levels were >10% at day 15, >1% at the end of induction (TP1) and >0.1% at the end of consolidation (TP2). Patients were allocated to receive an allo-SCT in case of: (1) not complete remission (CR) after TP1; (2) day 33 FCM-MRD level >1%, and FCM-MRD level >0.1% at TP2; or (3) persistence of positive FCM-MRD (>0.01%) after high-risk blocks of chemotherapy.

Globally, irrespectively, of the protocol treatment, we used a sensitivity threshold of 0.01% in this study to try to reproduce the previously reported results in the FRALLE and GRAALL protocols (3, 28).

### Mutation Screening and CNA Analysis

We analyzed the hotspot regions reported for *NOTCH1* (exons 26, 27, 28, 34), *FBXW7* (exons 9 and 10), *PTEN* (exon 7), *NRAS* (exons 1 and 2), and *KRAS* (exons 1 and 2) by Sanger sequencing as previously described (25, 36). CNAs were screened by MLPA using the SALSA MLPA P383 T-ALL kit (MRC Holland, Amsterdam, The Netherlands), according to the manufacturer's instructions. We used the Coffalyser software v.140721.1958 for the analysis (MRC-Holland, Amsterdam, The Netherlands). The P383 T-ALL kit assesses alterations (deletions or amplifications) in transcription factors (*LEF1* and *MYB*), genes involved in signal transduction (*PTEN, NF1*, and *PTPN2*), in cell cycle (*CDKN2A, CDKN2B*, and *CASP8AP2*), in epigenetic regulation (*EZH2, SUZ12*, and *PHF6*), and identifies the *STIL-TAL1* and *NUP214-ABL1* fusion genes. Full experimental details are given in the Supplementary Methods.

### Outcome Analyses

To assess the predictive value of the conventional and new oncogenetic risk factors, we analyzed the prognostic impact of the *NFPR* mutational status, first addressing each gene individually, and then grouped as previously reported (20). We applied the GRAALL oncogenetic classifier (28), and grouped our patients into gLoR and gHiR to compare their outcome. We also analyzed our patients with the FRALLE score, combining the oncogenetic classifier by GRAALL group with WBC count ≥200 × 10^9^/L and the MRD levels at TP1 (3), plus an additional time point at TP2.

### Statistical Analysis

Statistical analyses were carried out using R software (37), considering all *p*-values lower than 0.05 to be statistically significant. Details are provided in Supporting Information.

### Ethical Aspects

The ethical issues are detailed in Supplementary Material.

## Results

### Molecular Findings and Association With Clinical Features at Diagnosis

Molecular findings are shown in Table 1 and Supplementary Figure 2. Sixty-four patients (35%) harbored *NOTCH1* mutations (*NOTCH1*^*mut*^), 45% at HD-N domain, 5% at HD-C domain and 9% at PEST domain. No mutations were found at TAD domain (Supplementary Figure 3). Out of *NOTCH1*^*mut*^ patients, eleven cases with HD-C mutations also had concomitant mutations at the PEST domain (*NOTCH1*^double^). *FBXW7* was mutated in 25 cases (13%), alone in 16 patients and combined with *NOTCH1*^*mut*^ in 9 cases. Overall, we observed *NOTCH1/FBXW7* mutations in 80/182 cases (44%), in line with previous reports, albeit in the lower range. *PTEN* abnormalities (mutation or major deletion, *PTEN*^abn^) were found in 30 patients (20%), including 9 with *NOTCH1/FBXW7* mutations. *K/N-RAS* mutations were identified in 14 patients (8%). Overall, 108 patients (57%) harbored at least one mutation/deletion in *NOTCH1, FBXW7, PTEN*, or *K/N-RAS*. The mutations included point mutations, insertions, deletions and indels (Supplementary Table 3 and Supplementary Figure 3). *CDKN2A* and *CDKN2B* were deleted in 109 out of the 160 tested cases (68%) and 95 patients (60%), respectively, including 89 homozygous deletions for both genes.

The association of *NOTCH1, FBXW7, PTEN*^abn^, and *K/N-RAS* status with clinical variables is shown in Supplementary Table 4. Globally, and in each protocol, *NOTCH1* mutations significantly associated with females, while *K-RAS* mutations associated with WBC count ≥200 × 10^9^/L.

We could classify 168 patients according to the previously reported oncogenetic stratifier (Table 1). Fifty-eight patients (34.5%) presented *NOTCH1/FBXW7* mutations without a *PTEN/RAS* mutation (*NF*^*mut*^*PR*^*wt*^) and were considered as gLoR; the remaining 110 cases (65.5%) were classified as gHiR. The gHiR group included 15 positive patients for both *NOTCH1/FBXW7* and *PTEN/RAS* mutations, 26 patients harboring *PTEN/RAS* mutations only and 69 patients with all genes in a wild-type status. The distribution of oncogenetic risk-groups between SHOP-2005 and SEHOP-PETHEMA-2013 cohorts was similar (Table 1).

### Patients' Outcome in Consecutive SEHOP Protocols

The outcome of the 142 assessable patients according to the therapeutic protocol is shown in Supplementary Figures 4, 5. The median follow-up of the patients was 7.7 years for SHOP-2005 and 2.8 years for SEHOP-PETHEMA-2013 cohort. Overall, we observed a non-significantly better outcome in the newest protocol: the 5-year overall survival (OS) was 85.9 ± 4.0 vs. 76.5 ± 5.9% (*p* = 0.22), and the disease-free survival (DFS) was 85.3 ± 4.1 vs. 70.5 ± 6.4% (*p* = 0.080) for SEHOP-PETHEMA-2013 and SHOP-2005 patients, respectively. Noticeably, the cumulative incidence of relapse (CIR) was not statistically different in both protocols (12.1 ± 3.8% vs. 18.1 ± 5.5%, *p* = 0.49).

### FCM-MRD Predicted Outcome in Consecutive SEHOP Protocols

FCM-MRD data in the SHOP-2005 cohort was available for 46 and 35 out of 51 patients at TP1 and TP2, respectively. At TP1, patients with MRD <0.01% (*n* = 33) showed a significantly better outcome than patients with MRD ≥ 0.01%, with a 5-year OS of 84.8 vs. 53.8%, a 5-year DFS of 78.7 vs. 46.2%, and a CIR of 12.6 vs. 38.5% (*p* = 0.019, *p* = 0.009, and *p* = 0.039, respectively). At TP2, with less data available, we observed only a borderline significant difference with worse OS in patients with FCM-MRD ≥ 0.01% (*p* = 0.050; Supplementary Figure 6).

In SEHOP-PETHEMA-2013 patients, FCM-MRD data was available in 84 and 62 out of 91 cases at TP1 and TP2, respectively. In contrast with the former protocol, FCM-MRD significantly impacted on outcome at TP2 (5-year DFS for MRD <0.01% 92.6 vs. 68.6% for MRD ≥ 0.01%, *p* = 0.026; Supplementary Figure 7), whereas at TP1 only impacted on OS with a borderline significance (*p* = 0.049).

### Oncogenetics Impact on Outcome

**Figure 1:**
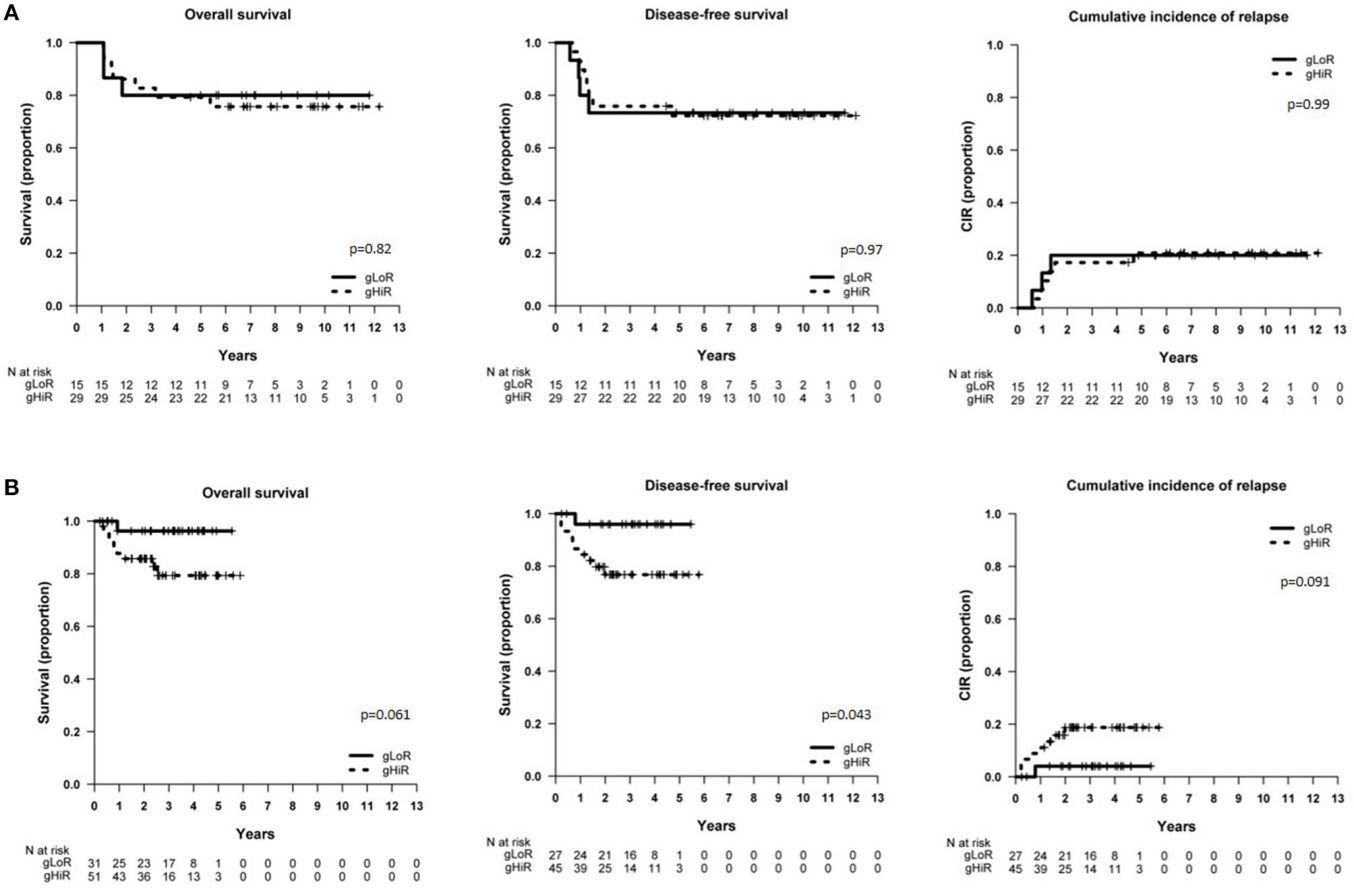
Survival of patients included in SHOP-2005 and SEHOP-PETHEMA-2013 protocol according to oncogenetics. **(A)** OS, DFS, and CIR in patients included in the SHOP-2005 protocol with gLoR *vs*. gHiR; **(B)** OS, DFS, and CIR in patients included in the SEHOP-PETHEMA-2013 protocol with gLoR *vs*. gHiR. gLoR: presence of NOTCH1 and FBXW7 mutations without lesions involving PTEN/RAS; gHiR: all the other genotypes.

In SHOP-2005 cohort, *NOTCH1* mutations did not significantly impact on outcome. Of note, all patients with *NOTCH1*^double^ mutations (*n* = 3) were alive in continuous CR (data not shown). Using the combination of the *NFPR* mutational status defined in the GRAAL oncogenetic stratifier (gLoR vs. gHiR), we observed no differences in patients'outcome (Figure 1A). In contrast, patients with *CDKN2A*/*B*^*homo*^ had a trend toward a worse DFS (*p* = 0.079) and a significantly higher CIR than patients with normal or heterozygous deletions of *CDKN2A/B* (*p* = 0.009, Supplementary Figure 8a).

In patients treated with SEHOP-PETHEMA-2013 protocol, those cases with *NOTCH1*^*mut*^ showed a better OS (*p* = 0.045), and all patients with *NOTCH1*^double^ mutations (*n* = 7) were alive in continuous CR (data not shown). In contrast to the former protocol, those SEHOP-PETHEMA-2013 patients classified as gLoR presented a significantly better DFS (*p* = 0.043), and a trend toward a better OS and a lower CIR (Figure 1B). Patients with *CDKN2A/B*^*homo*^ had a worse outcome, but the difference was not statistically significant (Supplementary Figure 8b).

Overall, we observed a different impact of oncogenetics on the SHOP-2005 and SEHOP-PETHEMA-2013 series of patients.

### Prognostic Impact of Classical Risk Factors

The univariate and multivariate analyses of OS and DFS for patients in SHOP-2005 and SEHOP-PETHEMA-2013 protocols is shown in Table 2. FCM-MRD was significantly predictive of outcome in both SHOP-2005 and SEHOP-PETHEMA-2013 cohorts (Supplementary Figures 6, 7), being statistically significant at early TP1 only for SHOP-2005 (*p* = 0.035) and at late TP2 only for SEHOP-PETHEMA 2013 protocol (*p* = 0.048). However, other classical risk factors impacted differently depending on the protocol: patients with hyperleukocytosis ≥200 × 10^9^/L showed a significantly worse DFS only in SHOP-2005 series. Sex, age, and CNS infiltration did not impact on outcome in either protocol.

**Table 2:** Univariate and multivariate analysis of OS and DFS for patients in SHOP-2005 and SEHOP-PETHEMA-2013 protocols.

	**SHOP-2005 protocol**				**SEHOP-PETHEMA-2013 protocol**			
	**Univariate analysis**		**Multivariate analysis**		**Univariate analysis**		**Multivariate analysis**	
	**HR [95% CI]**	***p***	**HR [95% CI]**	***p***	**HR [95% CI]**	***p***	**HR [95% CI]**	***p***
**OVERALL SURVIVAL (OS)**								
Age, years	0.99 [0.87; 1.12]	0.83	–	–	1.03 [0.89; 1.19]	0.67	–	–
**Sex**								
Female	Ref	Ref	–	–	Ref	Ref	–	–
Male	4.73 [0.61; 36.39]	0.14	–	–	1.71 [0.37; 7.90]	0.49	–	–
**WBC count**								
<200 × 10^9^/L	Ref	Ref	–	–	Ref	Ref	–	–
≥200 × 10^9^/L	2.00 [0.65; 6.11]	0.23	–	–	1.22 [0.32; 4.62]	0.77	–	–
**CNS involvement**								
Yes	Ref	Ref	–	–	Ref	Ref	–	–
No	0.38 [0.00; 2.41]	0.44	–	–	1.50 [0.42; 5.33]	0.53	–	–
**MRD TP1**								
<0.01%	Ref	Ref	–	–	Ref	Ref	–	–
≥0.01%	3.56 [1.14; 11.11]	**0.028**	–	–	3.51 [0.93; 13.29]	0.064	–	–
**MRD TP2**								
<0.01%	Ref	Ref	–	–	Ref	Ref	–	–
≥0.01%	3.80 [0.90; 16.00]	0.069	–	–	4.40 [0.80; 24.15]	0.088	–	–
**Oncogenetics**								
gLoR	Ref	Ref	–	–	Ref	Ref	–	–
gHiR	1.17 [0.30; 4.51]	0.82	–	–	5.73 [0.73; 45.27]	0.098	–	–
**DISEASE FREE SURVIVAL (DFS)**								
Age, Years	0.95 [0.84; 1.07]	0.38	–	–	1.09 [0.94; 1.26]	0.26	–	–
**Sex**								
Female	Ref	Ref	–	–	Ref	Ref	–	–
Male	5.89 [0.77; 44.82]	0.087			1.66 [0.36; 7.69]	0.52		
**WBC count**								
<200 × 10^9^/L	Ref	Ref	Ref	Ref	Ref	Ref	–	–
≥200 × 10^9^/L	2.88 [1.04; 7.96]	**0.042**	3.25 [1.12; 9.47]	**0.031**	1.33 [0.35; 5.02]	0.67		
**CNS involvement**								
Yes	Ref	Ref	–	–	Ref	Ref	–	–
No	0.33 [0.00; 2.41]	0.35	–	–	0.99 [0.26; 3.83]	0.99	–	–
**MRD TP1**								
<0.01%	Ref	Ref	Ref	Ref	Ref	Ref	–	–
≥0.01%	3.67 [1.28; 10.50]	**0.016**	3.15 [1.09; 9.18]	**0.035**	1.11 [0.29; 4.29]	0.88	–	–
**MRD TP2**								
<0.01%	Ref	Ref	–	–	Ref	Ref	Ref	Ref
≥0.01%	3.04 [0.76; 12.21]	0.12	–	–	5.59 [1.02; 30.70]	**0.048**	5.59 [1.02; 30.70]	**0.048**
**Oncogenetics**								
gLoR	Ref	Ref	–	–	Ref	Ref	–	–
gHiR	0.98 [0.29; 3.24]	0.97	–	–	6.37 [0.81; 49.76]	0.078	–	–

*HR, hazard ratio; CI, confidence interval; Ref, reference category; WBC, white blood cell; CNS, central nervous system; MRD, measurable residual disease; TP1, end of induction; TP2, end of consolidation; gLoR, genetic low-risk according to the oncogenetic stratifier; gHiR, genetic high-risk according to the oncogenetic stratifier. Bold values were considered significative as p < 0.05*.

### Use of a Clinical Prognostic Scores Combining Classical and New Risk Factors

**Figure 2:**
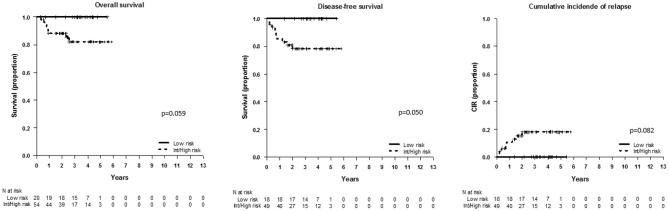
Outcome of patients included in SEHOP-PETHEMA-2013 according to the clinical classifier defined by Petit et al. (3). OS, DFS, and CIR in SEHOP-PETHEMA-2013 patients according to the FRALLE group combination, comparing the outcome of low-risk patients with the intermediate-risk and high-risk pooled together. The clinical classifier identified a subgroup of low-risk patients with excellent outcome, 100% OS at 5 years and no relapses. We observed a trend to the statistical significance in the outcome of the low-risk group vs. the remaining patients.

We used different combinations of classical and new risk factors to gain additional information on the relapse risk. First, we wanted to test in our series of patients the clinical classification reported by Petit et al. (3) integrating oncogenetics, MRD at TP1 (threshold 0.01%) and WBC count. By doing that, we observed a different distribution of subgroups in the analysis by protocols: in SHOP-2005, the outcome analysis following the clinical classification defined by the FRALLE group showed a poorer survival of high-risk patients as compared to the similar outcome of the low- and intermediate-risk patients (OS *p* = 0.25, DFS *p* = 0.024, and CIR *p* = 0.044; data not shown). In contrast, in SEHOP-PETHEMA-2013 patients, the low-risk patients had a better not significant outcome as compared to the survival of intermediate- and high-risk groups (*p* = 0.15, data not shown). We grouped the latter groups with similar outcome, and found that the clinical classifier was able to identify with borderline significance low-risk patients with 100% OS and no relapses (*p* = 0.059, *p* = 0.05, and *p* = 0.06 for OS, DFS, and CIR, respectively) (Figure 2).

Taken together, the oncogenetics, FCM-MRD at TP1 and WBC count, combined as defined by Petit et al. (3) in the FRALLE group, impacted significantly on outcome, and identified different risk populations in the analyzed cohorts.

## Discussion

Most pediatric T-ALL protocols only rely on MRD assessment to guide stratification in risk-groups, and robust and applicable risk-stratifying genetic factors at diagnosis are still lacking. The FRALLE group built a classifier based on WBC count, MRD at TP1 and an oncogenetic stratifier according to *NFPR* mutational status. However, some questions remain open regarding the FRALLE classifier: (1) would it be predictive of outcome in patients treated with BFM-based pediatric protocols?; (2) could MRD at TP2 be applied?, and (3) would this classifier be similarly predictive of patients' outcome when assessing MRD by FCM? To answer these questions, we analyzed a large cohort of pediatric T-ALL patients treated with consecutive Spanish protocols. We confirmed MRD as a prognostic risk-factor throughout different protocols, also when measured by flow-cytometry. However, oncogenetics impacted differently according to treatment, confirming that therapeutic modifications can lead to changes in the prognostic impact of biological variables like *NOTCH1* mutations.

We first evaluated the outcome of Spanish pediatric T-ALL patients in consecutive protocols. We observed a not statistically significant improvement in the 5-year DFS with SEHOP-PETHEMA-2013 protocol, as compared with the older SHOP-2005 protocol. The differences between both protocols could partly explain the different outcome. Briefly, both are BFM-inspired protocols, but in SEHOP-PETHEMA-2013 an induction IB course with cyclophosphamide, cytarabine, and mercaptopurine was incorporated. In this regard, an intensive induction and multiagent consolidation including cyclophosphamide is strongly recommended in T-ALL (38), and omitting the induction IB resulted in inferior outcome in large reported cohorts (39, 40). Also, a more intense asparaginase scheme including PEG-asparaginase was given in SEHOP-PETHEMA-2013. Thus, as compared to SHOP-2005 protocol, patients received increased doses of asparaginase and longer periods of asparagine depletion. The response to treatment was also differently assessed: in SEHOP-PETHEMA-2013 the prednisone response was added as stratifying criteria, and the non-centralized 4-colors FCM-MRD was replaced by 8-colors FCM-MRD with agreed panels, centralized in reference laboratories. Moreover, a lower number of patients underwent an allo-SCT in SEHOP-PETHEMA-2013 (Supplementary Figure 4), and had a lower non-leukemic mortality (data not shown). Overall, the intensification of the chemotherapy, a better stratification of patients and lower non-leukemic mortality in the latter protocol, could have contributed to the improvement of the survival in SEHOP-PETHEMA-2013 cohort. Similarly, a survival benefit dependent on mainly reducing toxicity alongside improving efficacy was recently described in adult T-ALL patients (41).

We then assessed the prognostic impact of conventional and new risk factors within each protocol. Hyperleukocytosis ≥200 × 10^9^/L was only predictive of a significantly worse DFS in SHOP-2005 cohort. Next, we analyzed the prognostic value of FCM-MRD in our cohorts. FCM-MRD levels impacted significantly on patients' outcome in both SHOP-2005 and SEHOP-PETHEMA-2013 protocols, proving the usefulness of FCM-MRD with a threshold of 0.01% to discriminate prognosis. This is especially relevant in the difficult setting of flow-cytometry assessment of MRD in T-ALL patients. However, FCM-MRD impacted differently according to the protocol and time point. The impact of MRD at late time points in T-ALL patients may vary between protocols, as reported by AIEOP-BFM and NOPHO Groups (6, 11). The discrepancies could be explained by differences in the treatment protocols, stratification criteria, cutoff levels and methods (11). In our study, FCM-MRD discriminated outcome both at early and late time points in SHOP-2005, whereas significant differences were observed in SEHOP-PETHEMA-2013 cohort at TP2. This could be driven by differences between both protocols in terms of intensity of treatment, with earlier stratification criteria using prednisone response, and early intensification therapy being added in SEHOP-PETHEMA-2013 patients, mimicking the backbone of current BFM protocols. Importantly, methodological differences in the FCM-MRD assessment between SHOP-2005 and SEHOP-PETHEMA-2013 can play a role in the results. Thus, in the latter protocol, 8-color FCM-MRD centralized in reference laboratories, replaced the non-centralized 4-color FCM-MRD performed in SHOP-2005. The increased number of markers in SEHOP-PETHEMA-2013 should, theoretically, improve the accuracy of the analysis, but we did not investigate the concordance between FCM and other methods like IG-TR MRD. On the other hand, the sensitivity in the FCM studies performed in SEHOP-PETHEMA-2013 was expected to be higher than in SHOP-2005 due to a higher number of acquired cells, but this point has not been confirmed nor a straightforward comparison between methodologies has been performed. Globally, in the SEHOP-PETHEMA-2013 cohort, FCM-MRD reproduced previous remarks about the relevance of late time points in BFM-based protocols. Overall, although the number of patients in each protocol is low, and even considering the less controlled setting in the older protocols, FCM-MRD appeared as a robust risk-factor in our Spanish series of patients.

As reported, we confirmed in our cohorts recurrent mutations in pediatric T-ALL patients. We found alterations in *NOTCH1/FBXW7* genes in 44% of our patients. The incidence of *NOTCH1* mutated cases in our series is low. However, similar incidences have been published in other series, ranging from 23 to 70.8% (42, 43). Specifically, 23 and 40% of *NOTCH1* mutations were reported in Turkish (44) and Japanese cohorts of patients (45), respectively. *FBXW7* mutations also range from 8.6 to 30.8% in the literature. Overall, the frequency of *NFPR* mutations, and mutation rates of *CDKN2A/B* deletions, were in the range of previously reported incidences (42, 46–48). When we classified patients into gLoR and gHiR, patients were grouped in the same way in both protocols. However, we found a lower proportion of gLoR patients compared to the FRALLE group, in parallel to the lower incidence of *NOTCH1/FBXW7* mutations in our cohort. The highly variable frequency in *NOTCH1*/*FBXW7* mutations could be explained by different factors including bias in sample storage, ethnicity, methodological aspects, and the total number of patients.

Regarding oncogenetic impact, we first studied the impact of individual genetic alterations. *NOTCH1* mutations had a favorable clinical impact in SEHOP-PETHEMA-2013 cohort, and all patients with *NOTCH1*^double^ mutations were alive in continuous complete remission. Neither *PTEN* nor *K/N-RAS* mutations had a significant impact on outcome. As reported, the homozygous deletion of *CDKN2A/B* associated with higher CIR in SHOP-2005 patients. In this line, recent studies have shown that the lack of deletions involving *CDKN2A/ARF/CDKN2B* locus, combined with undetectable MRD (≤ 0.01%) values, allowed the identification of a subset of adult T-ALL patients with better OS in the absence of allo-SCT (31).

The analysis of the combination of oncogenetic abnormalities yielded different results according to the protocols. Hence, the application of the oncogenetic classifier (*NFPR*) to our cohorts showed no impact in SHOP-2005 patients and a trend to a better OS and CIR, and a significantly better DFS in SEHOP-PETHEMA-2013 patients. We next tested the clinical classification reported by the FRALLE group, integrating oncogenetics, MRD at TP1 (threshold 0.01%) and WBC count. The FRALLE classifier allowed to identify different subgroups of patients with statistically different outcome in the whole cohort of Spanish patients. In the newest protocol SEHOP-PETHEMA-2013, a very low-risk group could be segregated with this score. Thereby, we reproduced the results of the FRALLE group in different, BFM-inspired therapeutic protocols. However, the results in SHOP-2005 and SEHOP-PETHEMA-2013 analyzed individually varied, probably due to differences between protocols.

The FRALLE-2000 protocol differed in several aspects from SHOP-2005 and SEHOP-PETHEMA-2013. Briefly, FRALLE-2000 and SHOP-2005 had similar indications of cranial irradiation, given both prophylactically in a subgroup of patients and therapeutically in all T-ALL patients, whereas cranial irradiation was omitted in SEHOP-PETHEMA-2013. The FRALLE-2000 protocol had more cyclophosphamide and anthracycline cumulative doses than SHOP-2005. On the other hand, SEHOP-PETHEMA-2013 had a prolonged intensification with PEG-asparaginase compared with FRALLE-2000 and SHOP-2005 protocols. Taken together, we observed that, even in the setting of different protocols, the oncogenetics, FCM-MRD at TP1, and WBC count, may be useful to refine the risk-stratification in pediatric T-ALL patients. In BFM-based protocols, the FRALLE classifier allowed to identify a subgroup of patients with very low-risk, who could be considered as potential candidates to de-intensify treatment. Our current protocol, SEHOP-PETHEMA-2013, aims to intensify treatment, and no de-intensification is considered. Interestingly, following the FRALLE risk stratification, a quarter of our intermediate-risk patients (20/76, 26%) could be considered as candidates to receive less intense treatment, and be moved to a lower-risk group. Our results, if confirmed in larger series, could help to consider the option of selected de-intensification therapy in future clinical trials.

Some factors may limit our study. Centralization in reference laboratories and biobanking was not mandatory in former protocols. Also, to assess the possible impact of differences in the therapeutic regimes, we analyzed the outcome of each protocol separately. Hence, the low number of patients in each group may have limited the power of statistics and precluded the finding of significant results. Finally, the median follow-up of SEHOP-PETHEMA-2013 cohort is short (2.8 years), but it covers most of the expected T-ALL relapses.

In summary, we present a national collaborative study of clinical and genetic prognostic factors in a large series of T-ALL pediatric patients. Notably, FCM-MRD predicted outcome in both Spanish protocols, being more important in TP2 in SEHOP-PETHEMA-2013. We could reproduce the FRALLE group's results in our BFM-based SEHOP protocols, and observed the predictive value of the combination of FCM-MRD, WBC count and oncogenetics to predict outcome at TP1. This classifier allowed, in our current protocol SEHOP-PETHEMA-2013, to identify a low-risk subgroup of patients with excellent outcome. Further studies in the context of controlled clinical trials would be necessary to confirm if the de-intensification therapy of a selected low-risk group of patients would lead to the same good outcome. Our results provide data that could be clinically relevant, as may help to apply tailored risk-directed treatments to reduce both toxicity and relapse.

## Data Availability Statement

The original contributions presented in the study are included in the article/Supplementary Material, further inquiries can be directed to the corresponding author/s.

## Ethics Statement

The studies involving human participants were reviewed and approved by Comitè Ètic d'Investigació amb medicaments (CEIm) de la Fundació Sant Joan de Déu. Written informed consent to participate in this study was provided by the participants' legal guardian/next of kin.

## Author Contributions

NV-G and MC designed the study and MC supervised the project. NV-G, EE-C, and CV-G performed the molecular analyses of the study. NV-G and SP-J analyzed the data and performed the statistical analyses. NV-G and MC wrote the paper with the contribution of SP-J, MT, JD, and SR. MT, AJ-V, MO, ML, LA, JV, AM, MP, JS, CG-C, MG-C, EM-C, AE, MM-G, LM, MV, MG-M and MC diagnosed patients and performed the main biological characterization of samples. AC, AP, AJ-V, JMF, AL, JLF, IB, AMo, AM-Q, PG-G, APM, MG, RR, SP, RA, JD, SR and MR-O recruited patients. All authors read and approved the final version of the manuscript. Other centers and investigators belonging to the Biological Committee and other integrands of the Group of Leukemia of the Spanish Society of Pediatric Hematology and Oncology (SEHOP) contributing to this study are listed in the participant investigators.

## Participant Investigators

The authors thank the contribution of other clinicians and researchers from different centers integrated in the Group of Leukemia of the Spanish Society of Pediatric Hematology and Oncology (SEHOP), for collecting data, providing and caring for study patients: Hospital Sant Joan de Déu (Nuria Conde, Sandra Pont, Sara Montesdeoca, Ignacio Isola); Hospital Niño Jesús (Ana Castillo), Hospital Universitari i Politècnic La Fe (Inés Gomez-Seguí), Hospital Carlos Haya (Antonia Pascual); Hospital General de Alicante (Fabián Tarín, María Tasso); Hospital Virgen de la Arrixaca (María Victoria Martinez-Sánchez, José Antonio Campillo, Eduardo Ramos-Eibal, Esther Llinares, Ana María Galera, María del Mar Bermúdez); Hospital Vall d'Hebrón (Anna Varela-Magallón); Hospital Virgen de las Nieves (Francisco Ruiz-Cabello, Pilar Jiménez, Mª José Ortega); Hospital Reina Sofía (Carmen Martínez); Hospital Virgen del Rocío (José Antonio Pérez-Simón); Hospital Gregorio Marañón (Carolina Martínez-Laperche); Hospital Santa Creu i Sant Pau (Josep Nomdedéu); Hospital Virgen de la Salud (Nerea Domínguez); Hospital Maternoinfantil de Badajoz (María Dolores de la Maya, María Belén Moreno); Hospital Marqués de Valdecillas (Mónica López-Duarte), Complejo Hospitalario de Navarra (María Sagaseta de Ilurdoz); Hospital Virgen de la Macarena (David García, Helga Benítez, Ana Fernández-Teijeiro); Hospital Son Espases (Marta Bernués, Laia Ferrés, José Antonio Salinas).

## Conflict of Interest

MT reports travel and accommodation support from Novartis (outside the submitted work); travel and accommodation support from Jazz Pharma and Shire/Servier (outside the submitted work); and travel and accommodation support from Amgen (outside the submitted work). JLF is a consultant/advisory member for Amgen, Jazz Pharmaceuticals, and Novartis (outside the submitted work), receives honoraria for speaking at symposia from Amgen, Servier, Jazz Pharmaceuticals, and Pfizer (outside the submitted work) and support for attending symposia from Servier and Jazz Pharmaceuticals (outside the submitted work). AM discloses talk fees from Jazz Pharma and Shire/Servier outside the presented work and reports travel and accommodation support from Jazz Pharma, Shire/Servier, and Novartis (outside the submitted work). JD reports advisory board honorarium, speaker fees, and travel and accommodation support from Jazz Pharma and Shire/Servier; personal fees, advisory board honorarium, speaker fees, and travel and accommodation support from Novartis (outside the submitted work); advisory board honorarium, and travel and accommodation support from Amgen (outside the submitted work); advisory board honorarium, speaker fees, and travel and accommodation support from Celgene (outside the submitted work); advisory board honorarium, speaker fees, and travel and accommodation support from Sobi (outside the submitted work). SR reports advisory board honorarium, speaker fees, and travel and accommodation support from Jazz Pharma and Shire/Servier; personal fees, advisory board honorarium, speaker fees, and travel and accommodation support from Novartis (outside the submitted work); advisory board honorarium, and travel and accommodation support from Amgen (outside the submitted work); advisory board honorarium, speaker fees, and travel and accommodation support from Celgene (outside the submitted work). MC discloses talk fees from Shire/Servier outside the presented work. The remaining authors declare that the research was conducted in the absence of any commercial or financial relationships that could be construed as a potential conflict of interest.
