# Supplementary material for: Measurable Residual Disease Assessed by Flow-Cytometry Is a Stable Prognostic Factor for Pediatric T-Cell Acute Lymphoblastic Leukemia in Consecutive SEHOP Protocols Whereas the Impact of Oncogenetics Depends on Treatment
Source: Front Pediatr. 2021 Feb 5;8:614521. doi: 10.3389/fped.2020.614521 (PMC7892614; doi:10.3389/fped.2020.614521)
Supplement: Supplementary file 1 [file Data_Sheet_1.docx]

Supplementary Material

[1 Supplementary Methods 2](#_Toc57279818)

[2 Supplementary Tables 7](#_Toc57279825)

[Supplementary Table 1. Main clinic and biological characteristics of the 189 pediatric patients with T-cell acute leukemia included in the molecular study. 7](#_Toc57279826)

[Supplementary Table 2. Comparison of the main characteristics of the SHOP-2005 and SEHOP-PETHEMA-2013 patients included in the present study (n=51, and n=91, respectively), *vs*. patients from the SHOP-2005 and SEHOP-PETHEMA-2013 protocols (n=82 and n=117, respectively) that did not enter the study. 8](#_Toc57279827)

[Supplementary Table 3. Full annotation of the mutations in *NOTCH1, FBXW7, PTEN, K-RAS* and *N-RAS* identified in the cohort of 189 analyzed patients. 9](#_Toc57279828)

[Supplementary Table 4. Association between genetic alterations and clinic-hematological features in the 189 pediatric patients with T-ALL with available sample and data at diagnosis 13](#_Toc57279829)

[3. Supplementary Figures 14](#_Toc57279830)

[Supplementary Figure 1. Flowchart of subjects and study design. 14](#_Toc57279831)

[Supplementary Figure 3. Graphical representation showing mutation distribution by domains in *NOTCH1, FBXW7, PTEN, NRAS* and *KRAS* gene. 16](#_Toc57279832)

[Supplementary Figure 4. Outcome of patients according to treatment protocol 17](#_Toc57279833)

[Supplementary Figure 5. Survival of the 142 patients included in SHOP-2005 and SEHOP-PETHEMA-2013 protocols. 18](#_Toc57279834)

[Supplementary Figure 6. Outcome of SHOP-2005 patients according to FCM-MRD at TP1 and TP2.. 19](#_Toc57279835)

[Supplementary Figure 7. Outcome of SEHOP-PETHEMA-2013 patients according to FCM-MRD at TP1 and TP2. 20](#_Toc57279837)

[Supplementary Figure 8. Survival of patients included in SHOP-2005 and SEHOP-PETHEMA-2013 protocol according to oncogenetics 21](#_Toc57279838)

[Supplementary Figure 9. Survival of patients included in SHOP-2005 and SEHOP-PETHEMA-2013 protocol according to *CDKN2A/B* status 22](#_Toc57279839)

[3 References 23](#_Toc57279842)

# Supplementary Methods

## Treatment and MRD monitoring in the SEHOP and PETHEMA guidelines

The SHOP 2005 and SEHOP-PETHEMA-2013 are two consecutive therapeutic protocols BFM (Berlin-Frankfurt-Münster)-inspired. The former has been previously described (Rives *et al*, 2012). In SEHOP-PETHEMA-2013 protocol, all T-ALL patients received a 4 drug induction therapy (IA: prednisone (60 mg/m^2^ days 1-28, vincristine 1.5 mg/m^2^ days 1, 15, 22 and 28, daunorubicin 30 mg/m^2^ days 1, 15, 22 and 28, PEG-asparaginase (PEG-ASP, 1,000 IU/m^2^ days 12 and 26) and triple intrathecal therapy (TIT). After induction IA, all patients received consolidation therapy with protocol IB (mercaptopurine, cyclophosphamide, cytarabine and TIT). After IB, intermediate-risk patients (IR) received consolidation therapy with high dose methotrexate (5g/m^2^ in 24h) and mercaptopurine, reinduction therapy (with dexamethasone, vincristine, doxorubicin, PEG-ASP, cyclophosphamide, mercaptopurine and cytarabine), followed by maintenance therapy with mercaptopurine and methotrexate without pulses for a total duration of therapy of 2 years. High Risk (HR) patients received as consolidation therapy high-risk blocks of chemotherapy, followed by a three reinduction phase and maintenance. Bone-marrow aspirates were taken on days 0, 15, and 29 of therapy, and additionally on day 79 (IR patients) or before each high-risk block until MRD was undetectable (HR patients). Patients with T-ALL were not eligible for standard-risk group stratification. Patients were stratified into HR therapy if poor prednisone response (PPR, >1,000 blasts/µL after one week of prednisone), poor early response (day 15 of induction bone marrow >25% blasts by morphology assessment (M3) and >10% blasts by FCM) or end of induction (TP1) M2 (5-25% blasts) or M3 bone marrow or FCM-MRD level >1% or >0.1% at the end of consolidation (TP2). Patients were stratified to allogeneic stem cell transplantation (allo-SCT) if not in CR after TP1, or day 33 MRD level >1%, or MRD level >0.1% at TP2.

## Immunophenotyping of T-ALL at diagnosis and FCM-MRD

SHOP-2005 protocol

MRD was measured by FCM using 4-color panels for identification and monitoring of leukemia-associated immunophenotypes (LAIPs) according to guidelines of group EGIL. At diagnosis, the FCM assays included monoclonal antibodies (mAbs) against the following markers described to identify LAIPs: CD79a, CD3, IgM, TdT and MPO (cytoplasmic/nuclear), CD10, CD20, CD19, CD33, CD13, CD117, CD22, CD45, CD66, CD8, CD4, CD3, CD5, CD7, CD38 and HLA-DR. For residual disease analysis, either patient-tailored antibody combinations or those employed at diagnosis were used. In this protocol, MRD levels >0.1% were considered positive and considered for clinical decisions. However, lower values were reviewed and analyzed in our study.

SEHOP-PETHEMA-2013 protocol

At diagnosis, 8-color panel according to EuroFlow consortium protocols was recommended. The antibody panels included: CD45, CD34, cyCD79a, CD19, cyCD3, sCD3, CD7, cyMPO, CD38, HLA-DR, nucTdT, CD10, CD5, CD2, CD1a, CD4, CD8, CD56, TCRαβ, TCRγδ, CD117, CD33, CD13.

For MRD detection down to the 0.01% level (with a required resolution of at least 20 events to refer a sample as positive), an optimum of 500000 nucleated cells had to be acquired. The combination of mAbs per tube was based on a backbone of these four mAbs: CD7, cytCD3, sCD3 y CD45. At least two tubes (combinations) were performed; the first tube was the same for all patients, and the second tube was performed based on the immunophenotype of the leukemic blasts at diagnosis.

| Tube | PacB/ V450 | PacO/V500/ KrO | FITC | PE | PerCP-Cy5.5/ PC5.5 | PC7 | APC | APCH7/ APC-AF750 |
| --- | --- | --- | --- | --- | --- | --- | --- | --- |
| 1 | cyCD3 | CD45 | cyTdT | CD1a | CD34 | CD2 | CD7 | sCD3 |
| 2 | cyCD3 | CD45 | X | X | X | X | CD7 | sCD3 |

X: optional

Myeloid: CD13 (PE, PC5.5, PerCP-Cy5.5, PC7); CD33 (PE, PC5.5, PC7, APC-AF750, V450); CD117 (PE, PC5.5, PerCP-Cy5.5, PC7)

Other: CD4 (FITC, PE, PerCP-Cy5.5, PC5.5, PC7, PacB, V450); CD8 (FITC, PE, PC7, PacB, V450), CD99 (PE)

## DNA and RNA extraction and reverse transcription

Bone marrow or peripheral blood samples from diagnosis were used for the analyses. Separation of mononuclear cells was performed using a density gradient with Ficoll-Hypaque (Sigma, St Louis MO, USA). Genomic DNA was extracted with QiaAMP DNA Blood and Gentragene DNA extraction kits (Qiagen, Hilden, Germany).

## Mutation screening and copy number alteration analysis

Details on the genes and methods used for the molecular analysis of the study are provided in Table 1. *NOTCH1, FBXW7, PTEN, KRAS* and *NRAS* hotspots were screened by Sanger sequencing. A 25 µl PCR mixture containing 0.3 units of HotStarTaq® (Qiagen, Hilden, Germany), PCR Buffer, 50 mM MgCl2, 5 mM deoxyribonucleotide triphosphates (dNTPs) (Qiagen, Hilden, Germany), 10 mM of forward and reverse primer, and 50 ng of gDNA/cDNA as a template was used. Cycling conditions were polymerase activation at 94^o^C for 5 min, following 40 cycles of 94^o^C for 1 minute, 60^o^C for 1 minute, and 72^o^C for 1 minute, followed by a final extension of 10 minutes at 72ºC. A nested PCR was performed for *NOTCH1* exons. The oligonucleotides are listed in Table 2.

All PCR products were purified, and Sanger sequencing was performed using a BigDye Terminator v3.1 Cycle Sequencing Kit (Applied Biosystems, Carlsbad, CA) in a 3,500 Genetic Analyzer (Applied Biosystems). The analyses were performed with Chromas 2.6.6 software, comparing electropherograms with the reference sequences accessed from the National Center for Biotechnology Information (NCBI): NOTCH1 (NG_007458.1; NM_017617.3), FBXW7 (NM_1013415.1; NG_029466.1), NRAS (NM_002524.4; NG_007572.1) and KRAS (NG_7524.1; NM_004985.4).

**Table 1.** Genes and methodology used for the molecular analysis.

| Gene | Chromosomal band | Exons | Method |
| --- | --- | --- | --- |
| *NOTCH1* | 9q34 | 26, 27, 34 | Sanger sequencing |
| *FBXW7* | 4q31 | 9, 10 | Sanger sequencing, |
| *PTEN* | 10q23.31 | 1*, 7, 9* | Sanger sequencing, *MLPA |
| *NRAS* | 1p13.2 | 2, 3 | Sanger sequencing |
| *KRAS* | 12p12.1 | 2, 3 | Sanger sequencing |
| *SIL* | 1p33 | 1, 6, 12 | MLPA |
| *TAL1* | 1p33 | 3, 6 | MLPA |
| *LEF1* | 4p25 | 1, 4, 7, 13 | MLPA |
| *CASP8AP2* | 6q15 | 1, 2, 6, 10 | MLPA |
| *MYB* | 6q23.3 | 2, 6, 16 | MLPA |
| *EZH2* | 7q36.1 | 4, 15, 21 | MLPA |
| *CDKN2A* | 9p21.3 | 2, 4 | MLPA |
| *CDKN2B* | 9p21.3 | 2 | MLPA |
| *MLLT3* | 9p21.3 | 1, 7 | MLPA |
| *NUP214* | 9q34.12-q13 | 2, 23 | MLPA |
| *ABL1* | 9q34.12-q13 | 4, 12 | MLPA |
| *LMO1* | 11p15.4 | 2, 4 | MLPA |
| *LMO2* | 11p13 | 1, 5 | MLPA |
| *NF1* | 17q11.2 | 26, 58 | MLPA |
| *SUZ12* | 17q11.2 | 10, 15 | MLPA |
| *PTPN2* | 18p11.21 | 1, 2, 4, 9 | MLPA |
| *PHF6* | Xq26.2 | 1, 3, 7, 11 | MLPA |
| *RAG2* | 11p12 | 1, 3 | MLPA |
| *MTAP* | 9q21.3 | 1 | MLPA |

**Table 2.** Oligonucleotides used to assess the mutational status of *NOTCH1, FBXW7, PTEN, KRAS* and *NRAS.*

| Primer | Exon | Forward 5'-3’ | Reverse 5'-3’ |
| --- | --- | --- | --- |
| *NOTCH1* gene |  |  |  |
| HD-N1 | 26 | AGCCCCCTGTACGACCAGTA | CTTGCGCAGCTCCTCCTC |
| HD-N1 nested | 26 | GACCAGTACTGCAAGGACCA | TCCTCGCGGCCGTAGTAG |
| HD-N2 | 26 | GTGCTGCACACCAACGTG | GAGGGCCCAGGAGAGTTG |
| HD-N2 nested | 26 | GCACACGGCCAGCAGATGAT | CGCCGGGTCTCACTCAC |
| HD-C | 27 | GTGGCGTCATGGGCCTCA | TAGCAACTGGCACAAACAGC |
| HD-C nested | 27 | CATGGGCCTCAGTGTCCT | GCACAAACAGCCAGCGTGT |
| TAD | 34 | GCAGCATGGCATGGTAGG | AACATGTGTTTTAAAAAGGCTCCT |
| PEST1 nested | 34 | AAACATCCAGCAGCAGCAAA | CACAGGCGAGGAGTAGCTGTG |
| PEST2 nested | 34 | GTGACCGCAGCCCAGTTC | AAAGGAAGCCGGGGTCT |
| *FBXW7* gene |  |  |  |
| FBXW7 E9 | 9 | TCTACCCAAAAGTAATCATCTTAAGTG | ATAGACGAACAAGTCCCAACCAT |
| FBXW7 E10 | 10 | GTTTTTCTGTTTCTCCCTCTGCA | ACCTTATGATTCATCAGGAGAGC |
| *PTEN* gene |  |  |  |
| PTEN E7 | 7 | GCTTGAGATCAAGATTGCAGATACAG | GTCTCACCAATGCCAGAGTAAGCA |
| *NRAS* gene |  |  |  |
| NRAS E1 | 1 | GACTGAGTACAAACTGGTGG | TGCATAACTGAATGTATACCC |
| NRAS E2 | 2 | CAAGTGGTTATAGATGGTGAAACC | AAGATCATCCTTTCAGAGAAAATAAT |
| *KRAS gene* |  |  |  |
| KRAS E1 | 1 | GGTGAGTTTGTATTAAAAGGTACTGGTG | CCTGTATTGTTGGATCATATTCGTCC |
| KRAS E2 | 2 | GGATTCCTACAGGAAGCAAGTAGTAA | CTATAATGGTGAATATCTTCAAATGATTTAGT |

## Statistical analysis

Statistical analyses were carried out using R software (R Core Team, 2019), considering all p-values lower than 0.05 to be statistically significant. Qualitative variables were described using absolute frequencies and percentages, whereas median, minimum and maximum were used to describe quantitative variables. To compare qualitative variables among groups, we used X2 or Fisher exact tests. To compare quantitative variables among groups, we used the Mann-Whitney U test. Event-free survival (EFS) was defined as the time from diagnosis until the first occurrence of refractoriness, relapse or death. Disease-free survival (DFS) was defined as the time from CR achievement until the date of relapse or death free of relapse. Overall survival (OS) was defined as the time from diagnosis until death. Patients who had not experienced an event were censored at the time of the last contact. Survival was estimated using the Kaplan-Meier estimator(Kaplan & Meier, 1958) and differences in survival between groups were assessed using the log-rank test or Gray test depending on the presence of competitive events (Greenwood, 1926; Kalbfleisch & Prentice, 1980). Cumulative incidence of relapse (CIR) only included patients that achieved CR and was estimated with relapse as an event of interest while taking into account death in first CR as a competing risk. CIR was compared among groups using the Gray test(Gray RJ, 1988). In the univariate analyses, we included the main clinical and biological prognostic factors, such as age, leukocytes, CNS involvement, phenotype and MRD at TP1 and TP2. The factors statistically significant in the univariate analyses were included in the multivariate analyses. Univariate and multivariate survival analyses were performed using the Cox proportional hazards model, except for CIR, where we used cause-specific hazard Cox models. Hazard ratios and their corresponding confidence intervals were derived from those models (Cox, 1972).  When Cox regression did not converge due to the absence of events, we applied Firth's penalised maximum likelihood bias reduction method (Heinze & Schemper, 2001; D, 1993).

## Ethic issues

The study was conducted in accordance with the ethical standards and the Declaration of Helsinki following the national and international guidelines and was approved by the authors' Local Ethics Committee. All samples were stored in the legally competent Biobank of each participant institution and were used after informed consent was obtained either from the patients or their legal guardians. All samples and data transfer were performed strictly following the national data protection regulations.

# Supplementary Tables

## Supplementary Table 1. Main clinic and biological characteristics of the 189 pediatric patients with T-cell acute leukemia included in the molecular study.

| Patients | Total no. (%)  189 |
| --- | --- |
| Sex (n=189)  Male  Female | 139 (73.5)  50 (26.5) |
| Age, years (n=188)  Median (range)  <10 years  ≥10 years | 8.0 (0.75-19.9)  113 (60.1)  75 (39.9) |
| WBC count, x10^9^/L, median (range) (n=187)  <200  ≥200 | 70 (1.0-897.0)  142 (75.9)  45 (24.1) |
| Blasts, median (range)  Bone marrow (n=145)  Peripheral blood (n=111) | 89.0 (25-100)  75 (0-100) |
| CNS (n=179)  CNS-1  CNS-2  CNS-3 | 137 (76.5)  18 (10.1)  24 (13.4) |
| Immunophenotype (n=137)  Pro-T  Pre-T  Early T-cell Precursor  Cortical  Mature  Other | 5 (3.7)  26 (18.9)  22 (16.1)  69 (50.4)  13 (9.5)  2 (1.4) |
| Protocol (n=189)  SHOP-2005  PETHEMA  SEHOP-PETHEMA-2013 | 51 (30.2)  41 (21.7)  91 (48.1) |
| Median follow-up, years (range) |  |
| Global series | 4.1 (0.02-17.2) |
| SHOP-2005  SEHOP-PETHEMA-2013 | 7.7 (4.6-17.0)  2.80 (0.3-5.6) |
| Overall Survival at 5 years, estimate ± SE, %  Global series  SHOP-2005  SEHOP-PETHEMA-2013  Event Free Survival at 5 years, estimate ± SE, %  Global series  SHOP-2005  SEHOP-PETHEMA-2013 | 81.9±3.5%  76.5±6%  85.9±4%  77.8±3.9%  70.5±6%  84.2±4% |

WBC: white blood cell. CNS: central nervous system. SE: Standard Error.

## Supplementary Table 2. Comparison of the main characteristics of the SHOP-2005 and SEHOP-PETHEMA-2013 patients included in the present study (n=51, and n=91, respectively), *vs*. patients from the SHOP-2005 and SEHOP-PETHEMA-2013 protocols (n=82 and n=117, respectively) that did not enter the study.

| **Patients** | **Vega-Garcia et al.**  **SHOP-2005**  **N=51** | **Entire cohort**  **SHOP-2005**  **N=82** | **p-value** | **Vega-Garcia et al.**  **SEHOP-PETHEMA 2013**  **N=91** | **Entire cohort**  **SEHOP-PETHEMA 2013**  **N=117** | **p-value** |
| --- | --- | --- | --- | --- | --- | --- |
| Sex  Male  Female | 38 (74.5%)  13 (25.5%) | 56 (68.3%)  26 (31.7%) | 0.57 | 67 (73.6%)  24 (26.4%) | 85 (72.6%)  32 (27.4%) | 1.00 |
| Age, years  Median (range)  <10 years  ≥10 years | 8.72 [1.69;19.9]  29 (56.9%)  22 (43.1%) | 10.3 [1.19;18.6]  40 (48.8%)  42 (51.2%) | 0.47 | 7.5 [1.4;16.0]  60 (65.9%)  31 (34.1%) | 8.6 [0.6;17.7]  76 (65.0%)  41 (35.0%) | 0.66  1.00 |
| WBC count, x10^9^/L, median  <200  ≥200 | 56.9 [1.0;675.0]  37 (72.5%)  14 (27.5%) | 68.0 [1.0;751.0]  62 (75.6%)  20 (24.4%) | 0.62  0.85 | 72.2 [1.9;897.0]  67 (75.3%)  22 (24.7%) | 78.0 [2.1;643.0]  87 (75.0%)  29 (25.0%) | 0.70  1.00 |
| Blasts, median  Bone marrow  Peripheral blood | 91.0 [25.0;100.0]  80.0 [0.0;98.0] | 89.0 [26.0;100.0]  75.0 [0.0;100.0] | 0.15  0.23 | 87.0 [34.0;100.0]  70.5 [0.0;100.0] | 84.3 [20.0;100.0]  67.0 [0.0;100.0] | 0.61  0.24 |
| CNS  CNS-1  CNS-2-3 | 47 (92.2%)  4 (7.8%) | 72 (90.0%)  8 (10.0%) | 0.76 | 55 (66.3%)  28 (33.7%) | 80 (69.0%)  36 (31.0%) | 0.80 |
| Median follow-up, years (range) | 7.7 [4.6;12.2] (n=38) | 6.1 [0.2;10.5] (n=54) | 0.001 | 2.8 [0.2;5.9] (n=80) | 1.7 [0.1;6.2] (n=98) | <0.001 |
| Overall Survival at 5 years, estimate ± SE, %  Event-Free Survival at 5 years, estimate ± SE, %  Disease-Free Survival at 5 years, estimate ± SE, % | 76.5%±5.9% (n=51)  75.3%±6.5% (n=51)  75.2%±6.5% (n=51) | 66.4%±5.5% (n=79)  71.3%±5.5% (n=79)  71.9%±6.5% (n=61) | 0.27  0.46  0.59 | 85.9%±4.0% (n=91)  85.8%±4.2% (n=81)  86.8%±4.1% (n=79) | 77.1%±5.6% (n=114)  75.8%±5.5% (n=115)  78.7%±5.8% (n=100) | 0.26  0.18  0.38 |

##

## Supplementary Table 3. Full annotation of the mutations in *NOTCH1, FBXW7, PTEN, K-RAS* and *N-RAS* identified in the cohort of 189 analyzed patients.

| Patient | Gene | Type | Nucleotide | Protein |
| --- | --- | --- | --- | --- |
| 1 | *NOTCH1* | missense | 4799T>C | Leu1600Pro |
|  | *FBXW7* | missense | 1393C>T | Arg465Cys |
|  | *NRAS* | missense | 35G>T | Gly12Val |
| 2 | *NOTCH1* | missense | 4754 T>C | Leu1585Pro |
|  | *FBXW7* | missense | 1393C>T | Arg465Cys |
|  | *NRAS* | missense | 35G>A | Gly12Asp |
| 3 | *NOTCH1* | missense | 4757G >C | Arg1586Pro |
|  | *FBXW7* | missense | 1268G>T | Gly423Val |
| 4 | *NOTCH1* | missense | 4754 T>A | Leu1585Gln |
|  | *FBXW7* | missense | 1555T>G | Tyr519Asp |
| 5 | *NOTCH1* | unknown | unknown | unknown |
|  | *FBXW7* | unknown | unknown | unknown |
| 6 | *NOTCH1* | unknown | unknown | unknown |
|  | *FBXW7* | unknown | unknown | unknown |
| 7 | *NOTCH1* | missense | 4799T>C | Leu1600Gln |
|  | *FBXW7* | missense | 1393C>G | Arg465Gly |
| 8 | *NOTCH1* | missense | 4778T>C | Leu1593Pro |
|  | *FBXW7* | missense | 1393C>T | Arg465Cys |
| 9 | *NOTCH1* | frameshift | unknown | unknown |
|  | *FBXW7* | missense | 1513C>T | Arg505Ser |
| 10 | *NOTCH1* | missense | 5033T>C | Leu1678Pro |
| 11 | *NOTCH1* | frameshift | 4818_4820delins15 | Phe1606fs |
|  | *PTEN* | frameshift | 696_696insA | Arg233fs*10 |
| 12 | *NOTCH1* | missense | 4721T>C | Leu1574Pro |
| 13 | *NOTCH1* | missense | 5033T>C | Leu1678Pro |
| 14 | *NOTCH1* | unknown | unknown | unknown |
| 15 | *NOTCH1* | missense | 4778T>C | Leu1593Pro |
|  | *NOTCH1* | missense | 7560G>A | Trp2520* |
| 16 | *NOTCH1* | missense | 7400 C>A | Ser2467* |
| 17 | *NOTCH1* | frameshift | 4818_4821delinsGTCCAAC | Phe1606fs |
| 18 | *NOTCH1* | missense | 5039T>A | Ile1680Asn |
| 19 | *NOTCH1* | missense | 4778T>C | Leu1593Pro |
|  | *NOTCH1* | frameshift | 7592_7537delTTCCTCACCCC | Phe2508fs*3 |
| 20 | *NOTCH1* | missense | 4778T>C | Leu1593Pro |
|  | *NOTCH1* | missense | 4790G>T | Ser1597Ile |
| 21 | *NOTCH1* | missense | 4778T>C | Leu1593Pro |
| 22 | *NOTCH1* | missense | 4719C>T | Thr1573Met |
|  | *NOTCH1* | missense | 4754T>C | Leu1585Pro |
| 23 | *NOTCH1* | missense | 4847T>A | Ile1616Asn |
| 24 | *NOTCH1* | missense | 4754 T>A | Leu1585Gln |
| 25 | *NOTCH1* | missense | 5039T>G | Ile1680Ser |
|  | *NOTCH1* | missense | 7318C>T | Gln2440* |
| 26 | *NOTCH1* | missense | 4754T>C | Leu1585Pro |
|  | *NOTCH1* | missense | 7246C>T | Gln2416* |
| 27 | *NOTCH1* | frameshift | 7519_7520delACCCC | His2507fs*7 |
|  | *NOTCH1* | frameshift | 7545_7548delGTCCCCTGA | Glu2515fs*39 |
| 28 | *NOTCH1* | missense | 4799T>C | Leu1600Pro |
|  | *NOTCH1* | missense | 7246C>T | Gln2416* |
| 29 | *NOTCH1* | missense | 4793G >C | Arg1598Pro |
| 30 | *NOTCH1* | missense | 4799T>C | Leu1600Pro |
| 31 | *NOTCH1* | frameshift | 7519_7529delCACCCCTTCCT | His2507fs*5 |
| 32 | *NOTCH1* | missense | 4799T>C | Leu1600Pro |
| 33 | *NOTCH1* | frameshift | 7401_7402insGT | Leu2468fs*10 |
| 34 | *NOTCH1* | frameshift | 7313_7318delinsCTAATGAT | Ser2439fs*# |
| 35 | *NOTCH1* | In-frame deletion | 4738_4740delATG | Met1580delMet |
| 36 | *NOTCH1* | missense | 4787T>A | Leu1596His |
| 37 | *NOTCH1* | missense | 4827A>G | Gln1584Arg |
|  | *NOTCH1* | missense | 4793G>C | Arg1598Pro |
| 38 | *NOTCH1* | frameshift | 7392_7393insAG | Leu2464fs*14 |
| 39 | *NOTCH1* | frameshift | 4739_4740delinsGGGC | Pro1581fs |
| 40 | *NOTCH1* | missense | 4790 G>T | Ser1597Ile |
| 41 | *NOTCH1* | missense | 4721T>A | Leu1574Glu |
|  | *NOTCH1* | missense | 5039T>A | Ile1680Asn |
| 42 | *NOTCH1* | missense | 4721T>A | Leu1574Glu |
| 43 | *NOTCH1* | missense | 4793G>C | Arg1598Pro |
|  | *NRAS* | missense | 35G>A | Gly12Asp |
| 44 | *NOTCH1* | frameshift | 7390delCTGCCCACGTCGC | Leu2464fs*9 |
| 45 | *NOTCH1* | missense | 4793G>C | Arg1598Pro |
| 46 | *NOTCH1* | missense | 5033T>C | Leu1678Pro |
| 47 | *NOTCH1* | missense | 5033T>C | Leu1678Pro |
|  | *NRAS* | missense | 35G>T | Gly12Val |
| 48 | *NOTCH1* | missense | 4723G>C | Val1575Leu |
| 49 | *NOTCH1* | missense | 4721T>C | Leu1574Pro |
| 50 | *NOTCH1* | frameshift | 4894_4906indel | Lys1606fs |
| 51 | *NOTCH1* | missense | 4754 T>C | Leu1585Pro |
| 52 | *NOTCH1* | missense | 4775T>C | Phe1592Ser |
| 53 | *NOTCH1* | missense | 4778T>C | Leu1593Pro |
| 54 | *NOTCH1* | missense | 4754 T>C | Leu1585Pro |
| 55 | *NOTCH1* | missense | 4799T>C | Leu1600Pro |
| 56 | *NOTCH1* | missense | 4775T>C | Phe1592Ser |
| 57 | *NOTCH1* | missense | 5033T>C | Leu1678Pro |
| 58 | *NOTCH1* | missense | 4754 T>C | Leu1585Pro |
| 59 | *NOTCH1* | missense | 4778T>C | Leu1593Pro |
|  | *NOTCH1* | missense | 7507C>T | Glu2503* |
| 60 | *NOTCH1* | unknown | unknown | unknown |
| 61 | *NOTCH1* | In frame | 4856_4857insGAC | Arg1594Asp |
| 62 | *NOTCH1* | frameshift | 4894_4895insGGGGGATGT | Phe1606fs |
| 63 | *NOTCH1* | frameshift | 4732_4734delGTG | Val1578del |
| 64 | *NOTCH1* | frameshift | unknown | unknown |
| 65 | *FBXW7* | missense | 1393C>G | Arg465Gly |
| 66 | *FBXW7* | missense | 1513C>T | Arg505Cys |
|  | *NRAS* | missense | 35G>A | Gly12Asp |
| 67 | *FBXW7* | missense | 1393C>T | Arg465Cys |
| 68 | *FBXW7* | missense | 1393C>T | Arg465Cys |
| 69 | *FBXW7* | missense | 1393C>T | Arg465Cys |
| 70 | *FBXW7* | missense | 1393C>T | Arg465Cys |
| 71 | *FBXW7* | missense | 1393C>T | Arg465Cys |
| 72 | *FBXW7* | missense | 1507G>A | Arg503Thr |
| 73 | *FBXW7* | missense | 1394G>A | Arg465Cys |
| 74 | *FBXW7* | missense | 1393C>T | Arg465Cys |
| 75 | *FBXW7* | missense | 1393C>T | Arg465Cys |
| 76 | *FBXW7* | missense | 1393C>T | Arg465Cys |
| 77 | *FBXW7* | missense | 1393C>T | Arg465Cys |
| 78 | *FBXW7* | missense | 1394G>T | Arg465Leu |
| 79 | *FBXW7* | missense | 1393C>T | Arg465Cys |
| 80 | *FBXW7* | missense | 1513C>A | Arg505Ser |
| 81 | *PTEN* | frameshift | 737_738delinsAACC | Leu247fs*7 |
|  | *NRAS* | missense | 34G>T | Gly12Cys |
| 82 | *NRAS* | missense | 34G>T | Gly12Cys |
| 83 | *PTEN* | frameshift | 696_701delinsCCC | Arg233fs*23 |
| 86 | *PTEN* | frameshift | 739_740insACCG | Leu247fs*20 |
| 87 | *PTEN* | frameshift | 694_696delinsCCCAGGTCCGG | Arg233fs*26 |
| 88 | *PTEN* | frameshift | 696_706delinsTCCCCTAGAG | Arg233fs*12 |
| 91 | *PTEN* | unknown | unknown | unknown |
| 92 | *PTEN* | frameshift | 703_703insG | Glu235fs*8 |
| 93 | *PTEN* | frameshift | 738_739insCC | Pro246fs*11 |
| 94 | *PTEN* | unknown | unknown | unknown |
| 95 | *PTEN* | frameshift | 696_696insG | Arg233fs*10 |
|  | *KRAS* | missense | 91G>A | Glu31Lys |
| 96 | *PTEN* | unknown | unknown | unknown |
| 97 | *PTEN* | frameshift | 743_744insGGTCCGT | Leu248fs*12 |
| 99 | *PTEN* | unknown | unknown | unknown |
| 100 | *PTEN* | frameshift | 696_696insA | Arg233fs*10 |
| 103 | *PTEN* | frameshift | unknown | unknown |
| 104 | *NRAS* | missense | 35G>A | Gly12Asp |
| 105 | *NRAS* | missense | 35G>A | Gly12Asp |
| 106 | *NRAS* | missense | 35G>T | Gly12Cys |
| 107 | *NRAS* | missense | 35G>A | Gly12Asp |
| 108 | *KRAS* | missense | 38G>A | Gly13Ala |

## Supplementary Table 4. Association between genetic alterations and clinic-hematological features in the 189 pediatric patients with T-ALL with available sample and data at diagnosis.

|  | ***NOTCH1/FBXW7*** | | | ***PTEN*** | | | ***K/N-RAS*** | | |
| --- | --- | --- | --- | --- | --- | --- | --- | --- | --- |
|  | Mutated (%)  N=80 | Wild type (%)  N=100 | p-value | Abnormalities (%)  N=33 | Wild type (%)  N=129 | p-value | Mutated (%)  N=14 | Wild type (%)  N=172 | p-value |
| **Age, years**  <10  ≥10 | 48 (60.0)  32 (40) | 60 (60.0)  39 (39.4) | 1.00 | 20 (20.8)  13 (20.0) | 76 (79.2)  52 (80.0) | 1.00 | 10 (71.4)  4 (28.6) | 100 (58.5)  71 (41.5) | 0.51 |
| **Sex**  Female  Male | 27 (33.8.)  53 (66.2) | 19 (19.0)  81 (81) | **0.037** | 9 (20.9)  24 (202) | 34 (79.1)  95 (79.8) | 1.00 | 4 (28.6)  10 (71.4) | 45 (26.2)  127 (73.8) | 0.76 |
| **WBC count**  <200x10^9^/L  ≥200x10^9^/L | 60 (75.9)  19 (24.1) | 77 (77.8)  22 (22.2) | 0.91 | 25 (20.0)  7 (20.0) | 100 (80.0)  28 (80.0) | 1.00 | 11 (78.6)  3 (21.4) | 130 (76.5)  40 (23.5) | 1.00 |
| **CNS involvement**  CNS-1  CNS-2  CNS-3 | 63 (84.0)  5 (6.67)  7 (9.33) | 68 (71.6)  10 (10.5)  17 (17.9) | 0.15 | 22 (19.1)  1 (6.67)  7 (31.8) | 93 (80.9)  14 (93.3)  15 (68.2) | 0.20 | 11 (78.6)  3 (21.4)  0 (0) | 123 (75.9))  15 (9.26)  24 (14.8) | 0.091 |
| **MRD at day +15**  <10%  ≥10% | 52 (82.5)  11 (17.5) | 54 (73.0)  20 (27.0) | 0.26 | 19 (19.0)  7 (28.0) | 81 (81.0)  18 (72.0) | 0.47 | 7 (87.5)  1 (12.5) | 101 (76.5))  31 (23.5) | 0.68 |
| **MRD at TP1**  <0.1%  ≥0.1% | 59 (85.5)  10 (14.5) | 66 (85.7)  11 (14.3) | 1.00 | 26 (21.8)  3 (18.8) | 93 (78.2)  13 (81.2) | 0.60 | 9 (90.0)  1 (10.0) | 121 (85.8)  20 (14.2) | 1.00 |
| **MRD at TP1**  <0.01%  ≥0.01% | 52 (75.4)  17 (24.6) | 50 (64.9)  27 (35.1) | 0.23 | 21 (21.0)  8 (22.9) | 79 (79.0)  27 (77.1) | 0.44 | 8 (80.0)  2 (20.0) | 98 (69.5)  43 (30.5) | 0.72 |
| **MRD at TP2**  <0.1%  ≥0.1% | 47 (92.2)  4 (7.8) | 51 (94.4)  3 (5.6) | 0.71 | 14 (15.7)  1 (20.0) | 75 (84.3)  4 (80.0) | 1.00 | 7 (100)  0 (0) | 94 (93.1)  7 (6.93) | 1.00 |
| **MRD at TP2**  <0.01%  ≥0.01% | 45 (88.2)  6 (11.8) | 47 (87.0)  7 (13.0) | 1.00 | 14 (16.5)  1 (11.1) | 71 (83.5)  8 (88.9) | 1.00 | 7 (100)  0 (0) | 87 (86.1)  14 (13.9) | 0.59 |

WBC: white blood cell. CNS: central nervous system. MRD: measurable residual disease. TP1: end of induction. TP2: end of consolidation.

# 3. Supplementary Figures

## Supplementary Figure 1. Flowchart of subjects and study design.


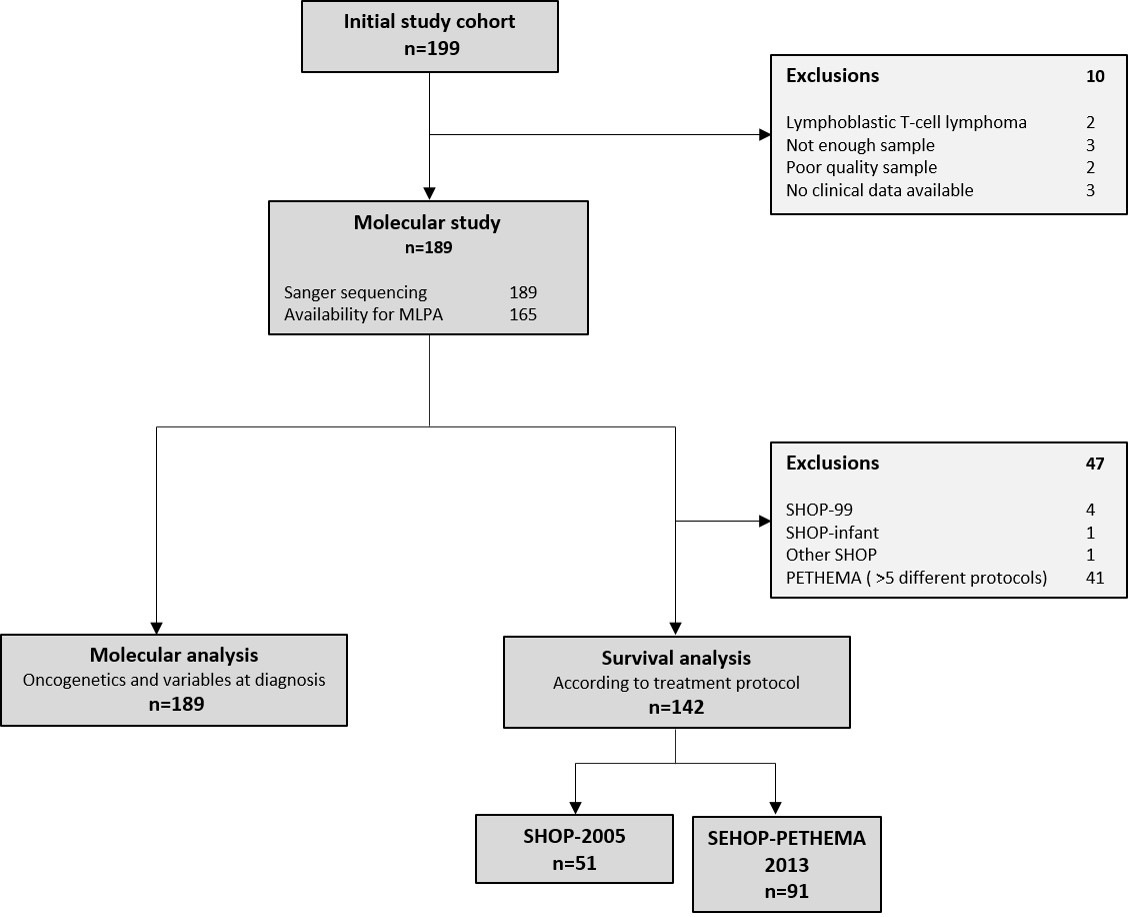


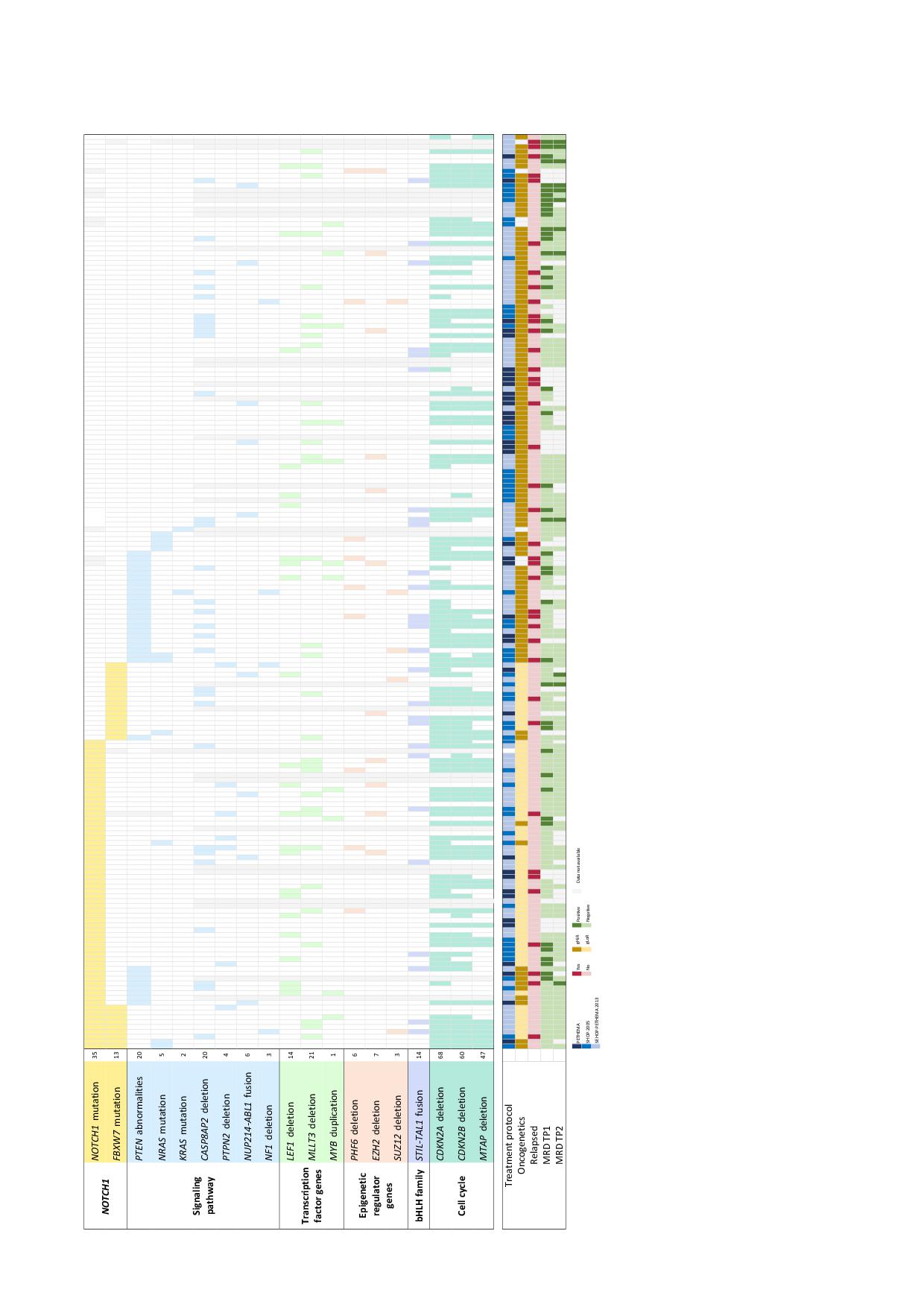
**Supplementary Figure 2. Mutational landscape of pediatric T-ALL alterations.** The percentage of mutation rates is shown. Genes are grouped according to their functional similarity or molecular pathways.

## Supplementary Figure 3. Graphical representation showing mutation distribution by domains in *NOTCH1, FBXW7, PTEN, NRAS* and *KRAS* gene.


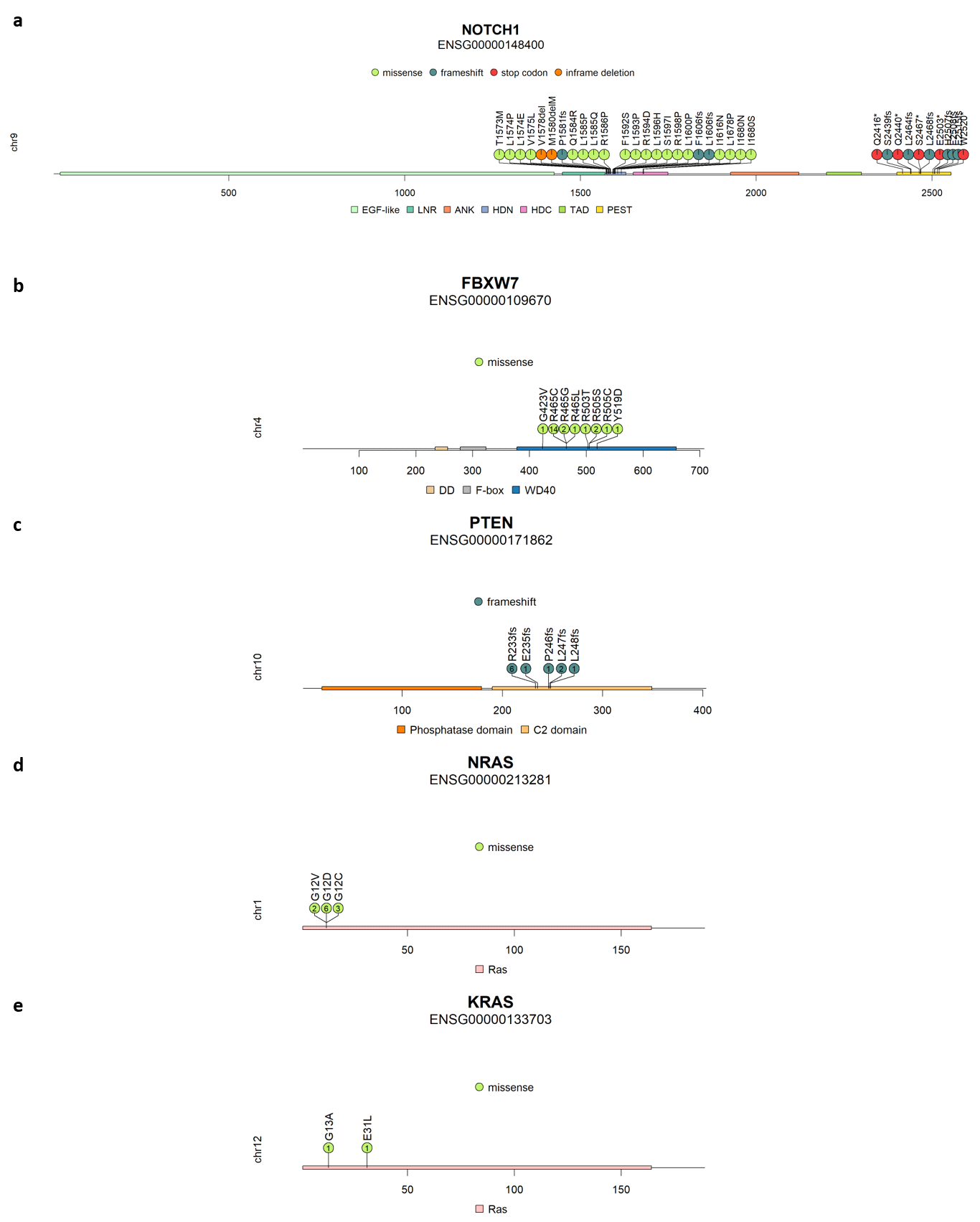


## Supplementary Figure 4. Outcome of patients according to treatment protocol. a) Flow-chart of the outcome of patients treated with SHOP-2005 protocol; b) Flow-chart of the outcome of patients treated with SEHOP-PETHEMA-2013 protocol.


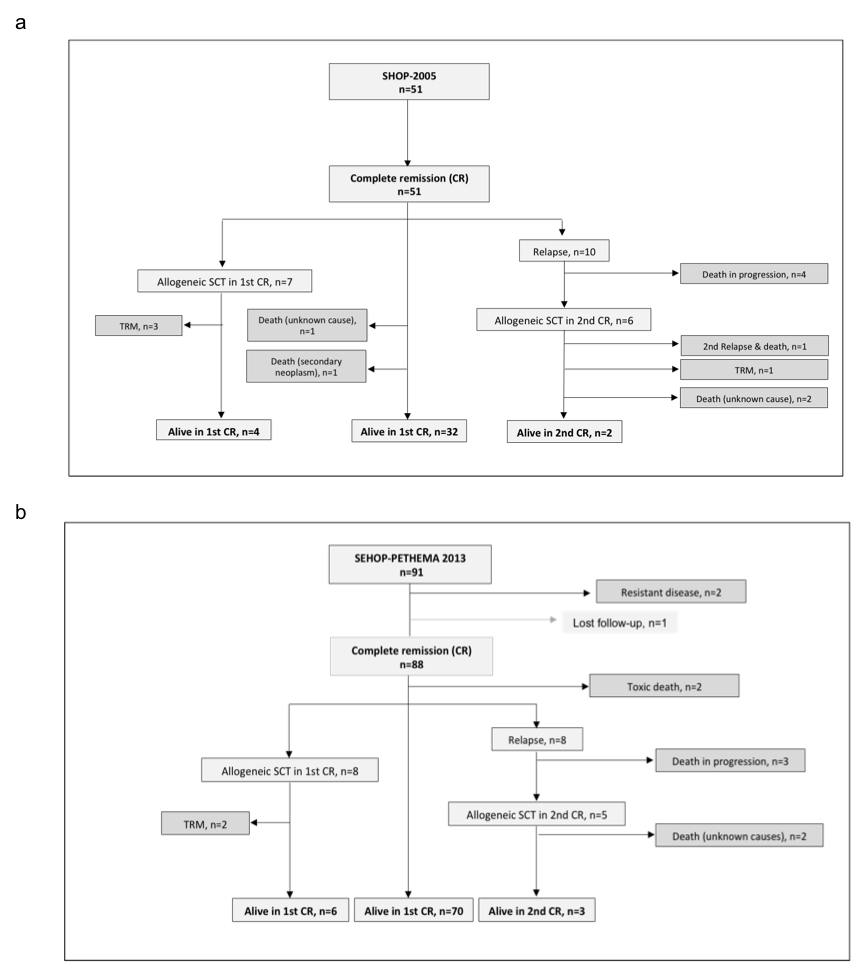


##
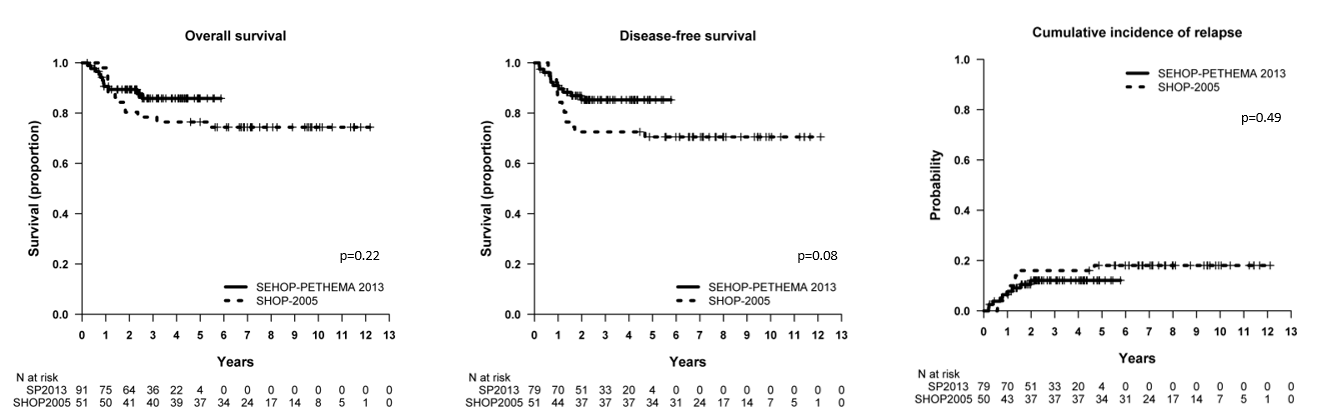
Supplementary Figure 5. Survival of the 142 patients included in SHOP-2005 and SEHOP-PETHEMA-2013 protocols.

|  | **Global**  **(n=142)** | | **SHOP-2005**  **(n=51)** | | **SEHOP-PETHEMA-2013**  **(n=91)** | | ***p-value**** |
| --- | --- | --- | --- | --- | --- | --- | --- |
|  | Estimate | 95%CI | Estimate | 95%CI | Estimate | 95%CI |  |
| Follow up | 4.1 (0.3-17.0) |  | 7.7 (4.6-17.0) |  | 2.80 (0.3-5.6) |  | **<0.001** |
| OS  5 years  10 years | 81.9 ± 3.5  79.9 ± 3.9 | 75.4-89.0  72.5-88.0 | 76.5 ± 5.9  74.4 ± 6.1 | 65.7-89.0  63.3-87.4 | 85.9 ± 4.0  - | 78.3-94.2  - | 0.22 |
| DFS  5 years  10 years | 78.3 ± 3.9  78.3 ± 3.9 | 71.0-86.4  71.0-86.4 | 70.5 ± 6.4  70.5 ± 6.4 | 59.1-84.2  59.1-84.2 | 85.3 ± 4.1  - | 77.6-93.8  - | 0.080 |
| CIR  5 years  10 years | 15.4 ± 3.5  15.4 ± 3.5 | 9.3-23.0  9.3-23.0 | 18.1 ± 5.5  18.1 ± 5.5 | 8.8 ± 29.9  8.8 ± 29.9 | 12.1 ± 3.8  - | 5.9 ± 20.7  - | 0.49 |

CI: confidence interval. OS: overall survival. EFS: event-free survival. DFS: disease-free survival. CIR: cumulative incidence of relapse.

## Supplementary Figure 6. Outcome of SHOP-2005 patients according to FCM-MRD at TP1 and TP2. a) OS, DFS and CIR in SHOP-2005 patients according to the FCM-MRD levels (<0.01% *vs.* ≥0.01%) at TP1; b) OS, DFS and CIR in SHOP-2005 patients according to the FCM-MRD levels (<0.01% *vs.* ≥0.01%) at TP2.

##
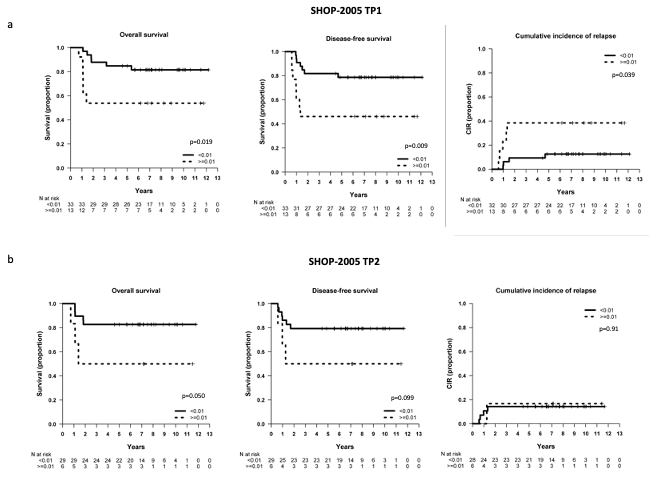


## Supplementary Figure 7. Outcome of SEHOP-PETHEMA-2013 patients according to FCM-MRD at TP1 and TP2. a) OS, DFS and CIR in SEHOP-PETHEMA-2013 patients according to the FCM-MRD levels (<0.01% *vs.* ≥0.01%) at TP1; b) OS, DFS and CIR in SEHOP-PETHEMA-2013 patients according to the FCM-MRD levels (<0.01% *vs.* ≥0.01%) at TP2.


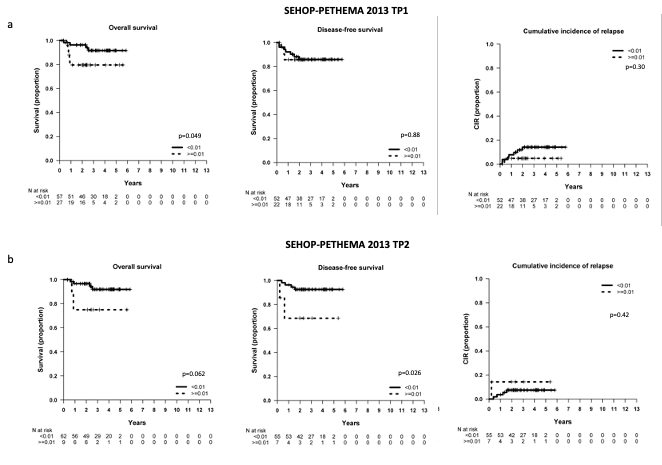


## Supplementary Figure 8. Survival of patients included in SHOP-2005 and SEHOP-PETHEMA-2013 protocol according to oncogenetics. a) OS, DFS and CIR in patients included in the SHOP-2005 protocol with gLoR *vs* gHiR; b) OS, DFS and CIR in patients included in the SEHOP-PETHEMA-2013 protocol with gLoR *vs* gHiR. *gLoR: presence of NOTCH1 and FBXW7 mutations without lesions involving PTEN/RAS; gHiR: all the other genotypes.*


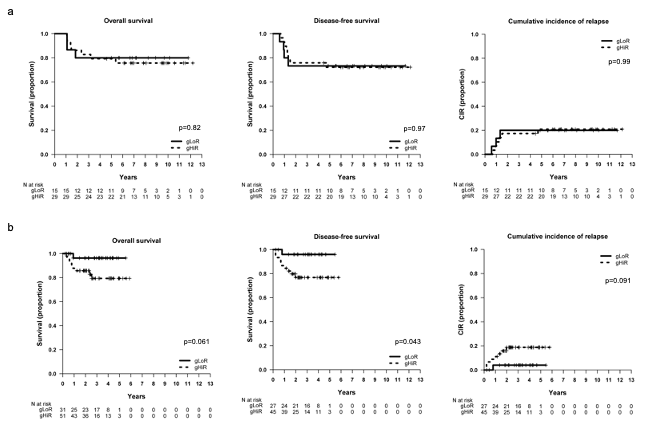


## Supplementary Figure 9. Survival of patients included in SHOP-2005 and SEHOP-PETHEMA-2013 protocol according to *CDKN2A/B* status. a) DFS and CIR in SHOP-2005 patients with *CDKN2A/B^homo^ vs* *CDKN2A/B* wild-type or with deletions in heterozygosis; b) DFS and CIR in SEHOP-PETHEMA-2013 patients with *CDKN2A/B^homo^* *vs* *CDKN2A/B* wild-type or with deletions in heterozygosis.


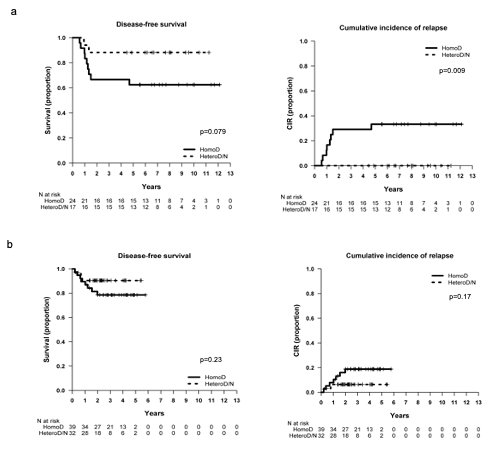


# References

Cox, D. (1972) Regression models and life-tables. *J R Stat Soc B*, 187–220.

D, F. (1993) Bias reduction of maximum likelihood estimates. *Biometrika*, 80:27–38.,.

Gray RJ (1988) A class of K-sample tests for comparing the cumulative incidence of a competing risk. *Ann Stat*, 16, 1141–1154.

Greenwood, M. (1926) The natural duration of cancer. *Rep Public Health Med Subj*, 1–26.

Heinze, G. & Schemper, M. (2001) A solution to the problem of monotone likelihood in Cox regression. *Biometrics*, 57, 114–9 Available at: http://www.ncbi.nlm.nih.gov/pubmed/11252585 [Accessed April 21, 2020].

Kalbfleisch, J. & Prentice, R. (1980)The statistical analysis of failure time data New York: John Wiley and Sons, Inc.

Kaplan, E. & Meier, P. (1958) Non parametric estimation from incomplete observations. *Journal of American Statistics Association*, 457–481.

R Core Team (2019) R: A language and environment for statistical computing. Available at: https://www.r-project.org/.

Rives, S., Estella, J., Camós, M., García-Miguel, P., Verdeguer, A., Couselo, J.M., Tasso, M., Molina, J., Gómez, P., Fernández-Delgado, R., Navajas, A., Badell, I. & grupo cooperativo SHOP (Sociedad Española de Hemato-Oncología Pediátrica) (2012) [T-cell pediatric acute lymphoblastic leukemia: analysis of survival and prognostic factors in 4 consecutive protocols of the Spanish cooperative study group SHOP]. *Medicina clinica*, 139, 141–9 Available at: http://www.ncbi.nlm.nih.gov/pubmed/22459573 [Accessed October 22, 2019].
